# Large-scale ordering of nanoparticles using viscoelastic shear processing

**DOI:** 10.1038/ncomms11661

**Published:** 2016-06-03

**Authors:** Qibin Zhao, Chris E. Finlayson, David R. E. Snoswell, Andrew Haines, Christian Schäfer, Peter Spahn, Goetz P. Hellmann, Andrei V. Petukhov, Lars Herrmann, Pierre Burdet, Paul A. Midgley, Simon Butler, Malcolm Mackley, Qixin Guo, Jeremy J. Baumberg

**Affiliations:** 1 Nanophotonics Centre, Cavendish Laboratory, Department of Physics, University of Cambridge, Cambridge CB3 0HE, UK; 2 Department of Physics, Prifysgol Aberystwyth University, Wales SY23 3BZ, UK; 3 Schlumberger Gould Research Center, Cambridge CB3 0EL, UK; 4 Deutsches Kunststoff-Institut (DKI), Darmstadt D-64289, Germany; 5 Van't Hoff Laboratory for Physical and Colloid Chemistry, Department of Chemistry, Debye Institute for Nanomaterials Science, Utrecht University, Utrecht 3584 CH, The Netherlands; 6 Department of Chemical Engineering and Chemistry, Laboratory of Physical Chemistry, Eindhoven University of Technology, Eindhoven 5600 MB, The Netherlands; 7 Department of Materials Science and Metallurgy, University of Cambridge, 27 Charles Babbage Road, Cambridge CB3 0FS, UK; 8 Department of Chemical Engineering and Biotechnology, University of Cambridge, Cambridge CB2 3RA, UK; 9 Department of Electrical and Electronic Engineering, Synchrotron Light Application Center, Saga University, Saga 840-8502, Japan

## Abstract

Despite the availability of elaborate varieties of nanoparticles, their assembly into regular superstructures and photonic materials remains challenging. Here we show how flexible films of stacked polymer nanoparticles can be directly assembled in a roll-to-roll process using a bending-induced oscillatory shear technique. For sub-micron spherical nanoparticles, this gives elastomeric photonic crystals termed polymer opals showing extremely strong tunable structural colour. With oscillatory strain amplitudes of 300%, crystallization initiates at the wall and develops quickly across the bulk within only five oscillations. The resulting structure of random hexagonal close-packed layers is improved by shearing bidirectionally, alternating between two in-plane directions. Our theoretical framework indicates how the reduction in shear viscosity with increasing order of each layer accounts for these results, even when diffusion is totally absent. This general principle of shear ordering in viscoelastic media opens the way to manufacturable photonic materials, and forms a generic tool for ordering nanoparticles.

## Abstract (web summary)

Packing nanoparticles into ordered superstructures finds applications in photonic materials, but fabrication over large scales is challenging. Zhao *et al*. show a roll-to-roll approach to prepare flexible films of ordered polymer nanoparticles via an oscillatory shear-induced structural transition.

## Graphical abstract

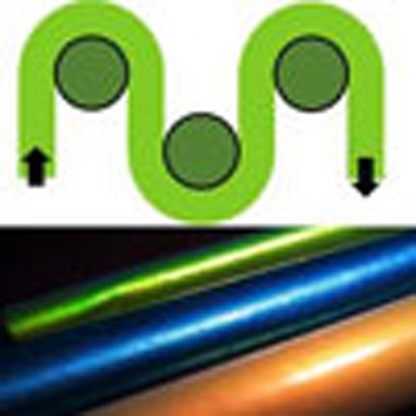

Nanostructural organization elicits useful material properties for a wide range of composites, ranging from colloids to granular systems. While extensive studies have focussed on nanoparticles equilibrated in low viscosity solvents^1^,^2^,^3^, these are transient and do not deliver permanently fixed architectures. Particulate-filled polymers, such as silica sphere reinforced resins, produce enhanced mechanical properties^4^,^5^,^6^, but particle concentrations are low and the packing order is not of concern. On the other hand, it is known that when high concentrations of particles are regularly packed, the system can exhibit interesting properties such as photonic bandgaps^7^,^8^, slow light propagation, or refractive index modification. More generally with increasing demand for flexible functional composites in wearable devices, sensors, electrodes and solar cells^9^,^10^,^11^, there is a strong need for progress in ordering dense particulate elastomers.

Shearing has long been known to induce transient order in solutions of colloidal particles, though excessive shear is found to break up any ordering^12^. Oscillatory shear has frequently been applied to colloidal and granular systems as a driving force for structural transitions^13^,^14^,^15^,^16^,^17^,^18^. Various transient ordered structures such as strings, sliding layers and crystallites have been generated in colloidal suspensions^19^, while crystals have also been induced by oscillatory shear in colloidal glasses and granular systems near the jamming point^20^,^21^, showing its potential for dense particulate assembly. More recent studies of the kinetics involved suggest that shearing induces banding with regions of high and low order, possessing different viscosities that are separated by a sharp boundary that can move^22^.

Photonic crystals constructed of spherical nanoparticles are excellent prototypical ordered superstructures as they give strong opaline structural colours from the multiple scattering of light off the periodic planes. Despite great endeavour, no manufacturing technique has yet been able to create continuous roll-to-roll opal sheets on any significant scale. We show how, unlike typical colloidal assembly routes, it is advantageous to remove all solvent to produce permanent nanostructures, which are locked in place, avoiding the phase separation of particles and solvent that quenches local annealing of sphere positions. Core-shell nanoparticles are thus employed to produce a ∼50 vol% dispersion phase of 200-nm-scale hard cross-linked spheres and a continuous phase of a gum-like medium, creating polymer opals (POs)^23^. These optical composites behave mechanically like rubbers and exhibit strain-sensitive colours due to Bragg diffraction from the opaline packing of the spheres^24^, leading to various promising applications^25^,^26^. It is important to recognize that POs are distinct from colloids and granular systems (Fig. 1). Colloids are entropy-driven systems comprised of sub-micron spheres exhibiting Brownian motion in low viscosity media such as water (*η*∼10^−3^ Pa·s)^14^,^27^,^28^. Granular systems are athermal gravity-driven suspensions with much larger spheres (>1 μm) in media such as air and slurry (approaching the jamming limit)^29^,^30^. In contrast, our POs are comprised of sub-micron polystyrene (PS) spheres in a gum-like polyethylacrylate (PEA) matrix. The PEA is bonded to each PS sphere as a shell and is viscoelastic above its glass transition temperature *T*_*g*_∼0 °C with *η*∼10^3^–10^4^ Pa·s at 100 °C (Supplementary Fig. 1). With a Reynolds number 10^6^ times smaller, and a characteristic Péclet number 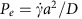 (where 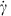 is the shear rate, *a* the particle radius and *D* the Stokes–Einstein diffusion coefficient) 10^6^ times larger than colloids, the PS sphere mobility is strongly inhibited and Brownian motion suppressed, precluding any self-assembly. On the other hand, the structural stabilization of particles by gravity-driven ‘bridging' as in granular systems^31^ is also absent. Instead, interactions in POs are dominated by inter-particle collisions and the matrix–particle elastic interactions that stick the spheres together, enabling the generation of non-equilibrium structures such as strings and sliding layers after shear.

We show how shearing such a highly viscoelastic composite (*E*∼1 MPa, *η*∼10^6^ Pa·s at low oscillation frequency, Supplementary Figs 1 and 2) provides strong forces for ordering in three dimensions at high speed. While some other ordering rheological systems (for example, colloidal glasses, gels) may exhibit a degree of viscoelasticity, as they are both viscous and elastic under deformation, the final PO structures here have a fully reversible stretch-tunability and mechanical stability/robustness, which is manifestly lacking in photonic crystals derived from either colloidal assembly or monolithic ‘top-down' methods. The key characteristics of POs in this context are: first, a high volume fraction, which is here above 50%; second, they are highly viscoelastic, with modulus some 10^5^ higher than the other cases described above; and third, very strong particle–medium interface bonding, with no continuous fluid medium. Our bending-induced oscillatory shear (BIOS) technique provides a controlled roll-to-roll process (for >100 m scale opal films), and allows us to study the mechanism of ordering as well as develop a general purpose model. We find that POs crystallize only with sufficient strain, *γ*≈300%, improving exponentially with the number of shear oscillations and reaching equilibrium after 40 oscillations. The resulting nanoarchitecture is predominantly randomly stacked hexagonal close-packed *r-hcp*, with *hcp* layers randomly shifted to one of the two possible hollow-site positions, which in turn redistributes some of the X-ray Bragg peaks into characteristic extended one-dimensional features. Compared with previous methods, this BIOS technique is a significant development for large-scale bulk manufacturing of ordered dense nanoparticle composites, is a generic approach, and can be generally applied to a very wide range of precursors (although requiring shell encapsulation).

## Results

### PO film fabrication and the mechanism of BIOS

**Figure 2:**
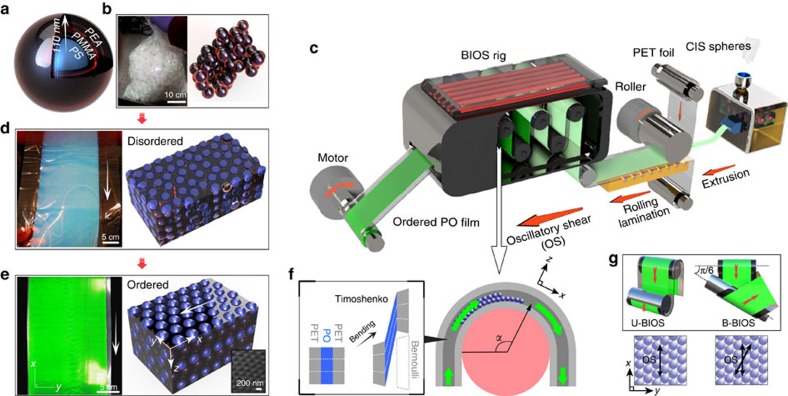
Fabrication of opal films and mechanism of BIOS. (**a**) PS-PMMA-PEA core-shell sphere. (**b**) Kg-scale core-shell spheres at room temperature after drying (left) leaves disordered spheres stuck together (right). (**c**) Production line of large-scale PO film with two types of BIOS. (**d**) The PET-PO-PET film after rolling-lamination (left), arrow indicates processing direction, with spheres packed randomly (right). (**e**) PO film after BIOS (left) with improved sphere packing (right), arrow indicates shear direction. Inset SEM image shows *hcp* packing at the surface. (**f**) Mechanism of BIOS inside the PET-PO-PET sandwich, dashed white lines show the bending-induced deformation in different layers. (**g**) Shear direction of U-BIOS (left) and B-BIOS (right), indicated by arrows.

Instead of simply blending PS spheres and PEA matrix (as they cannot be suitably mixed), POs are fabricated from PS-PMMA-PEA core-shell particles 223 nm in diameter (Fig. 2a), and volume ratio between the components of 34:10:56. The core-shell spheres are synthesized by emulsion polymerization (Fig. 2b)^26^,^32^, and put through our standard extrusion-rolling-lamination procedure for film production (Fig. 2d)^25^. The PEA shell softens when heated, and fills all the interstices between the spheres under stress. At this stage the films exhibit only faint colours from incoherent Mie scattering off the randomly packed low-index contrast spheres^33^. The PMMA interlayer covalently grafts the PEA chains to the PS spheres, providing strong particle–matrix interactions so that the spheres contribute to the resistance against stress^34^. Otherwise, as in template infiltration^35^, the weak bonding between spheres and matrix concentrates deformation energy in the matrix instead of onto the spheres which loses all crystallization (Supplementary Fig. 3)^36^,^37^.

**Figure 3:**
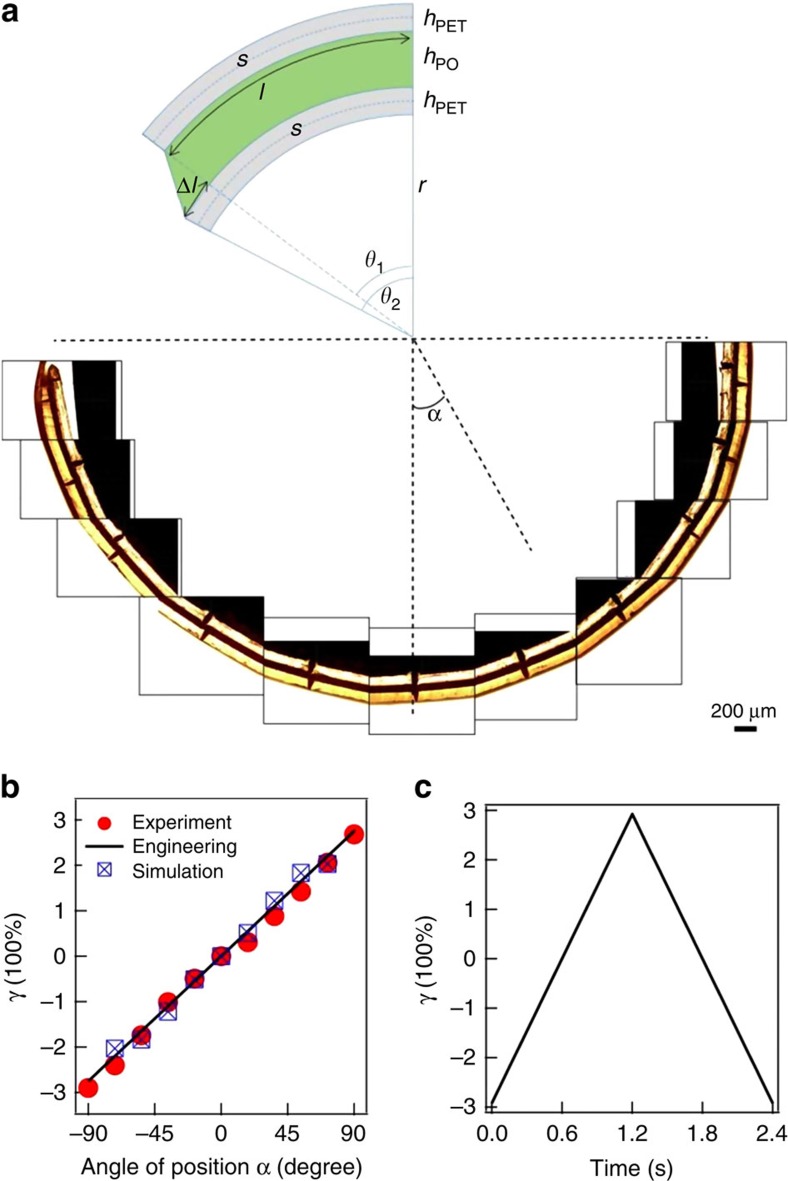
Schematic of sandwich theory. (**a**) Experimental measurements of the shear strain distribution after bending the sandwich composite film onto a cylinder, images taken with an optical microscope in bright field. A montage of images from different positions of the film is shown, with α being the position angle. The bright strips are PET foils, while the dark region in between is the PO film. The relative shifts between the marks on the top PET foil and the bottom PET foil indicate shear strain at different positions. The angular interval between the marks is ∼18°. (**b**) Experimental and Abaqus simulation results of γ distribution across the PO film with BIOS as a function of position angle (α) compared with the engineering approximation. Abaqus simulations performed with ratio between Young's modulus of PET and PO, *R*_*E*_=1,000, where result has been corrected for the true thickness in simulation. (**c**) Change of the shear strain inside the PO film within one oscillation, using an engineering approximation.

**Figure 4:**
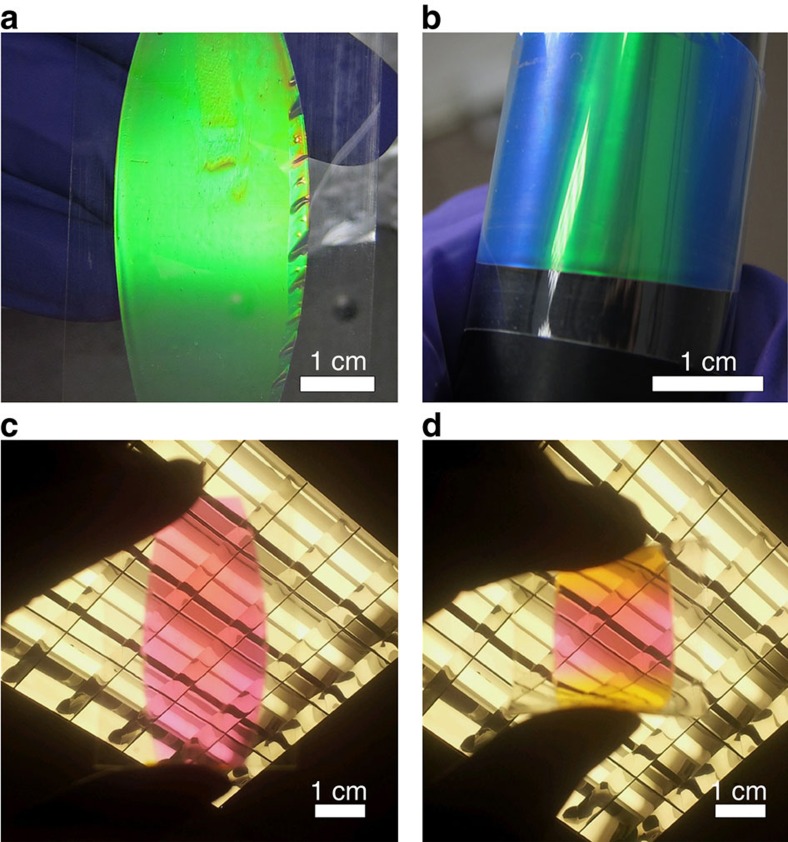
A green PO sample with 20 passes of U-BIOS. (**a**) Flat PO film between PET foils. (**b**) Bending the film onto a black cylinder, green colour in the middle part of the film remains unchanged to the eye. (**c**) Transmission colour of the flat film. (**d**) Transmission color of the film while bending, with the colour of the middle region remaining constant to the eye, indicating an unchanged layer spacing. Hence, there is no obvious normal strain inside the PO film along the thickness direction.

For the BIOS process, the *h*_PO_=80-μm-thick PO film is sandwiched between two *h*_PET_=120 μm polyethylene terephthalate (PET) sheets (Fig. 2c). The PET-PO-PET laminate constitutes a ‘sandwich' beam (according to linear sandwich theory, an extension of the Timoshenko linear beam theory), as the PET is far more rigid than the PO, with deformation occurring due to core shear (Supplementary Fig. 2)^38^,^39^. By bending this laminate around a cylinder, strong shear is created inside the PO purely parallel to the surface, as shown in the composite optical microscope image of the sample shear strain distribution after bending (Fig. 3) with a corresponding schematic representation in Fig. 2f (see also Fig. 4, Supplementary Movies 1 and 2). We take the angle at which the roller makes last contact with the laminate as *θ*_2_, with *s* being the unstretched length of PET film from the midpoint of roller contact and this last contact at *θ*_2_ (Fig. 3a). In this case, 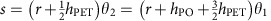, which gives


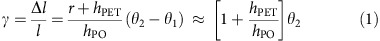


For the 180° bend of the PO film around the roller depicted, this gives *γ* increasing up to 300% in accordance with both experimental and Abaqus finite element analysis simulation results (Fig. 3b,c, Supplementary Discussions). As we discuss below, the direction of shear orients the close-packed lines of spheres in each *hcp* plane controlling the in-plane ordering. We thus employ two variants of the BIOS process in which either the shear direction through the rollers is maintained as unidirectional (U-BIOS), or we provide a bidirectional roller alignment (B-BIOS) where it is first sheared along the *x* direction and then along a 30° twisted direction (Fig. 2g). This allows us to investigate also the effects of shear direction on the anisotropy of the ordering.

Ordered PO films are obtained after this BIOS process, exhibiting bright structural colours (Figs 2e and 4) from Bragg diffraction at wavelength 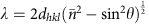, where *d*_*hkl*_ is the plane spacing, 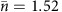 is the mean refractive index for POs, and *θ* is the incident angle. After BIOS, the spheres are found to be packed in *hcp* planes with the most close-packed direction parallel to the *x* direction (Fig. 2e, inset). The BIOS effects depends on shear strain *γ*, shear rate 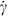 and temperature *T* (Supplementary Figs 5 and 6). Compared with colloids and granular systems where crystals are obtained with *γ*<100% (refs 40, 41), here crystallization of POs demands larger *γ* because sufficient shear stress is needed to shift particles well beyond the yield strain to facilitate their repositioning. This may be further seen in terms of the microscopic forces between particles in the specific case of POs; the elastic nature of the shells dictate that frictional contact forces between adjacent particles are extremely high. Therefore, small movements are accommodated by the elastic shell, without resulting in any permanent repositioning of the shell–shell boundaries. Above a critical strain (and shear), however, the frictional shell forces are overcome (see below). We use *T*=120 °C, *γ*=300% and 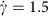 s^−1^ for the samples discussed below unless stated.

### BIOS-induced crystallization

Understanding how oscillatory shear works in the PO system to produce crystallization is daunting due to the complex interactions and challenging structural characterization. A detailed understanding is obtained here by combining optical spectroscopy and microscopy in *k*-space, focussed ion beam plus scanning electron microscopy (FIB-SEM) in real space^42^, small-angle X-ray scattering (SAXS) in reciprocal space^43^ and simulations.

**Figure 5:**
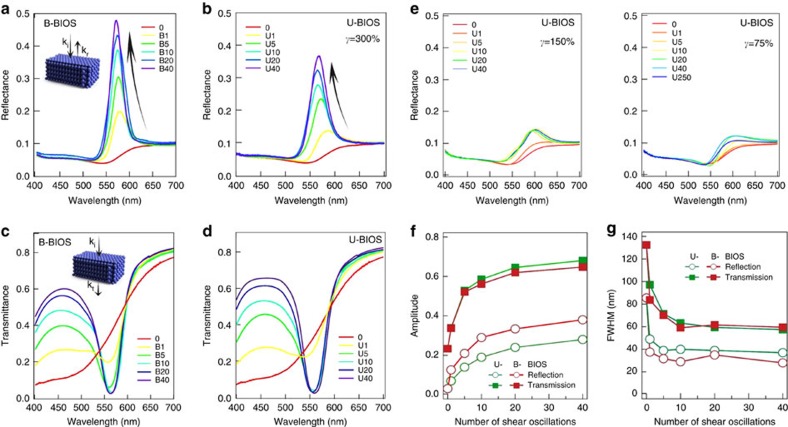
Normal incidence reflectance and transmittance of polymer opal films. (**a**) Reflectance of B-BIOS samples. (**b**) Reflectance of U-BIOS samples. (**c**) Transmittance of B-BIOS samples. (**d**) Transmittance of U-BIOS samples. (**e**) Reflectance of U-BIOS samples as in **b** but using BIOS with smaller strain amplitudes, *γ*=150% (left) and 75% (right). (**f**,**g**) Extracted peak amplitude and spectral linewidths. B*n* (U*n*) denotes samples with *n* passes of B-BIOS (U-BIOS).

Optical spectra demonstrate that crystallization occurs rapidly during BIOS. By measuring normal incidence reflection and transmission of PO samples (Fig. 5), we observe a steady opening of the photonic stopband with increasing passes of BIOS. In reflection, a Bragg diffraction peak appears immediately after one pass of U-BIOS and B-BIOS. The peak intensity increases and the full-width at half maximum (FWHM) decreases, both with exponential rates of 5±1 passes reaching full equilibrium after 40 passes (Fig. 5f,g). While the U-BIOS films saturate with resonant reflectivity near 0.4 at *λ*=565 nm and FWHM of 40 nm, the B-BIOS samples have larger reflectivities near 0.5, longer wavelength of 570 nm and narrower resonance of 30 nm indicating a slightly larger lattice spacing and greater order. Similar trends are found in transmission, with the B-BIOS dip at 562 nm red-shifted from the U-BIOS dip at 558 nm. However, in contrast to reflection, the U-BIOS transmission dip is now both deeper and sharper than that from B-BIOS. This suggests that the surface planes of the sample (of which 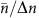
=20 layers are probed by reflection, dependent on refractive index contrast Δ*n*=0.11) are more ordered in B-BIOS, while the deep bulk of the sample that is probed in transmission is better ordered in U-BIOS. This is also supported by the better short wavelength transmission (around 450 nm) in U-BIOS. This trade-off between surface and bulk order will be accounted for below. The expected angle dependence of the Bragg peak in specular reflection is seen (Supplementary Figs 7–9) indicating the dominant layered ordering produced. The only evidence of additional bandgaps is seen at high angles along the *θ*_*y*_ direction in U-BIOS samples^44^,^45^, suggesting the presence of (200)-type planes from more three-dimensional (3D)-ordered *fcc* crystals (Supplementary Fig. 7). We confirm that optical properties of both the top and bottom surfaces of the films are identical.

Earlier reports have empirically confirmed that the efficacy of shear ordering of POs ceases below a certain strain value (see Finlayson *et al*. and Snoswell *et al*.)^25^,^46^. While more difficult to precisely quantify in these earlier experiments, this threshold level of strain clearly exceeds the critical yield strain (25%) beyond which the lattice planes in the opal are found to start slipping past each other (see Kontogeorgos *et al*.)^24^, and optimal processing strain values are consistently found to be >100%. Our experiment results, especially with regard to the optical properties, show significant improvements in ordering going from 75 to 150% up to 300% strain amplitudes (Fig. 5e,b).

**Figure 6:**
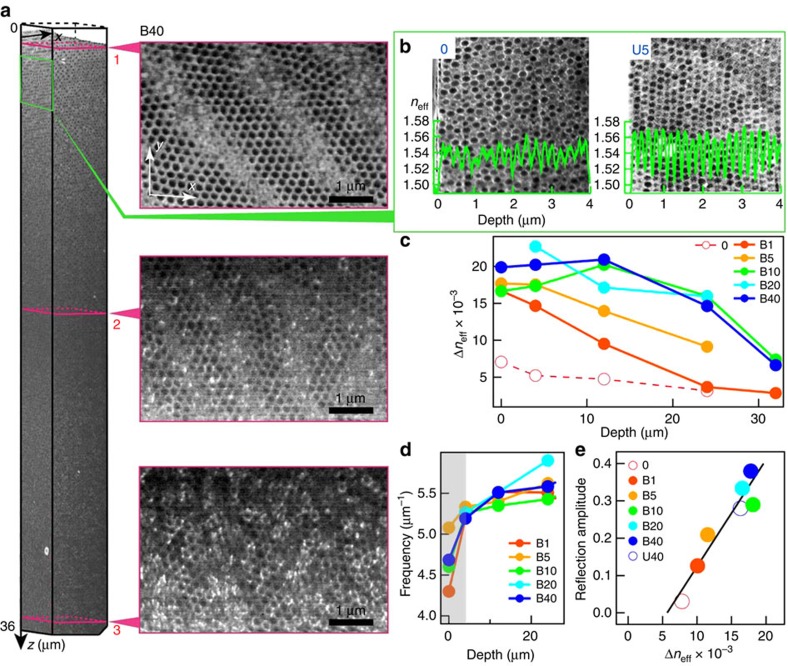
FIB-SEM characterization of sphere locations. (**a**) Reconstructed 3D structure of PO sample B40 with horizontal slices extracted at depths labelled. Cuts are slightly tilted to the *hcp* planes, giving images of several neighbouring layers separated by blurred stripes. (**b**) Cross-section SEM images of samples pre-BIOS (0), and U5 around different depths. Extracted *n*_eff_(*z*) variations (left axis) are superimposed (green). (**c**) Effective refractive index contrast Δ*n*_eff_ at different depths of B-BIOS samples. (**d**) Main spatial frequencies of *n*_eff_ at different depths in B-BIOS samples. (**e**) Linear relation between reflection amplitude and Δ*n*_eff_ of B-BIOS samples and U40.

We use FIB-SEM for characterising the real space structure of POs (Fig. 6). Samples are stained with RuO_4_ to enhance the electron density contrast (Supplementary Fig. 10). Slices in *xy* planes normal to the sample surface are successively cut with FIB, and imaged by *in situ* SEM. The 3D equilibrium structure of the sample with 40 passes of B-BIOS (B40) is reconstructed from many slices (Fig. 4a) allowing the in-plane organization parallel to the sample surface to be extracted at different depths (Fig. 5a). Near the surface, spheres pack in *hcp* planes with the close-packed spheres along *x*. Different orientations of the *hcp* crystal structures similar to multi-domains are also observed along with numerous dislocations. The packing order of the *hcp* planes degrades with depth, and the layers finally break into islands of *hcp* fragments at *z*>30 μm. We identify >200 well-ordered *hcp* layers after 40 passes, which theoretically should produce >90% Bragg reflection at normal incidence but the dislocations scatter a significant fraction of this light into a wider angular cone^25^.

Reconstructing cross-sections at different depths for different samples (Fig. 6b, Supplementary Figs 11–19, Supplementary Movie 4) and extracting the sphere positions allows the effective refractive index *n*_eff_(*z*) distribution with depth to be calculated. Both the main spatial frequency, which gives the average layer spacing over the area (*F*=*d*_*hkl*_^−1^) and the refractive contrast Δ*n*_eff_ (Fig. 6c,d, Supplementary Fig. 20) reveal details of the BIOS mechanism. Near the surface the initially disordered arrangement rapidly crystallizes, but with a spatial frequency *F*, which is 15% smaller in the surface layers compared with the bulk, emphasising the role of the nonlinear shear strain. We use the *n*_eff_(*z*) distribution to predict the optical properties using a transfer matrix model (Supplementary Fig. 21, Supplementary Discussions), which reveals that mainly the *hcp* layers between *z*=8–16 μm contribute to the reflection peak at *λ*=570 nm. We find excellent correlation (Fig. 5f) between the average Δ*n*_eff_(*z*=12–24 μm) for different samples and their corresponding measured reflectivity amplitude (with background subtracted), which agrees well with simulations and explains why reflection from B-BIOS is higher than U-BIOS (Fig. 5f). In some samples, the rapidly changing distribution of layer spacings near the surface can elicit double-peak reflection features from deep and shallow layers, which are seen in both predictions and experiment (Supplementary Figs 6 and 22).

**Figure 7:**
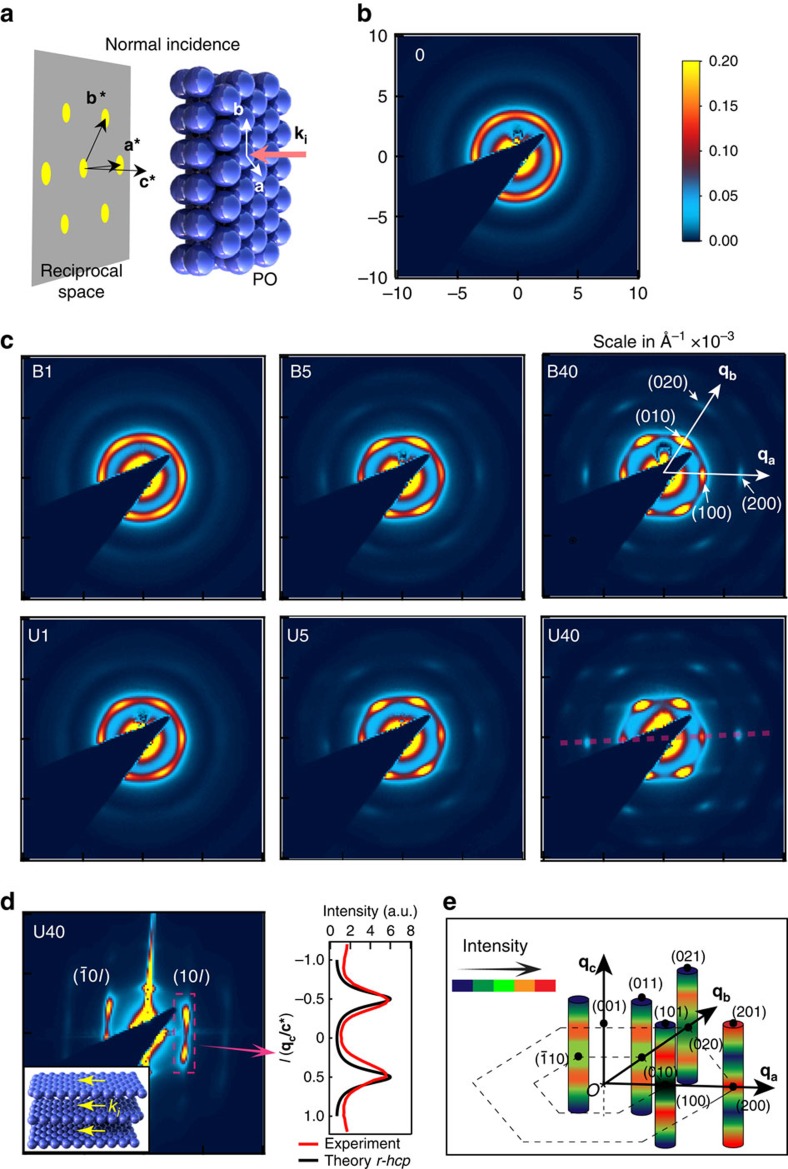
SAXS structure factor of BIOS-processed opals. (**a**) Illustration of SAXS at normal incidence with incident wave vector **k**_**i**_, unit vectors **a**,**b** of hexagonal lattice, a*, b* and c* are corresponding reciprocal lattice unit vectors. (**b**,**c**) SAXS patterns of different samples at normal incidence, with **q**_**a**,**b**_ along a*, b*, respectively. (**d**) Left, grazing incidence SAXS pattern (see inset geometry) showing scattering along selected rods on the red dashed line of sample U40 in **c**. Right, extracted scattering intensity along rod (10*l*) compared to scattering from *r-hcp* structure in theory. Separation between scattering peaks corresponds to layer spacing of 197 nm in real space (*F*=5.1 μm^−1^). (**e**) Intensity distributions along different rods, adjusting for the form factor. All SAXS images are (10 × 10) × 10^−3^ Å and on the same colour scale.

The average stacking and in-plane packing order of the *hcp* planes can be characterized with SAXS (relying on such large and uniform samples)^47^,^48^. Extracting the form factor from disordered samples, allows the SAXS scattering to be normalized. Indexing in the hexagonal coordinate system (Fig. 7a)^49^, the scattering intensity in reciprocal space is obtained for different samples at normal incidence (Fig. 7, Supplementary Figs 23 and 24). Before shearing, diffraction rings from the amorphous structures are seen, but these steadily organize into hexagonal patterns of diffraction spots with increasing BIOS passes, tracking the crystallization process and formation of *hcp* planes. The in-plane order can be assessed from the shapes of the spots, primarily from the (100) and (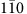) spots on the first-order ring, which are subject to detailed analysis (Supplementary Fig. 25). As expected, the FWHM of the spots in the azimuthal direction (long axis) *Λ*_*s*_ and in the radial direction (short axis) *Λ*_*r*_ both decrease exponentially with increasing passes of BIOS, indicating clear improvement of the *hcp* plane packing order. However, the results also show that U-BIOS induces better orientational order than B-BIOS, while B-BIOS induces better positional order in the *y* direction than U-BIOS. This indicates that U-BIOS creates more line dislocations with Burgers vector parallel to *y* while B-BIOS anneals these dislocations but orders the bulk less well. These line dislocations are confirmed by analysing the Bragg spot (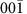) from optical scattering experiments (Supplementary Figs 26 and 27)^50^. Strong anisotropy is observed in the strength of spots on the same diffraction ring. The integrated intensity of the (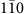) spots is much higher than those of (100) spots in both B-BIOS and U-BIOS samples, with a contrast increasing with more passes (and twice as strong in U-BIOS as in B-BIOS). The contrast is reversed for second-order spots (200) and (020). Using simulations^49^, we explain this anisotropy from a sliding layer model (Supplementary Figs 28–31) in which the *hcp* layers are shifted in both *x* and *y* directions from their ideal close-packed lattice registries. These shifts create different phases for the layers and strongly modulate scattering. The layers in U-BIOS and B-BIOS shift differently, thus resulting in different anisotropies in SAXS. Our models agree with the FIB-SEM results, which show that spheres of nearest layers are shifted away from the close-packed positions (Fig. 6).

The stacking order of the *hcp* layers is revealed by scattering along the Bragg rods in the ***q***_***z***_ direction (Supplementary Fig. 35). These rod diffraction patterns along (10*l*), (20*l*), (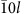) and (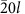) of a B40 sample at parallel incidence to the *hcp* planes (Fig. 7d,e) show good agreement with simulated predictions of *r-hcp* structures (Fig. 7d, black)^51^. Detailed analysis of the gonio-SAXS (Supplementary Figs 33–37, Supplementary Discussions) shows that differences in scattering between the rods ((10*l*) versus (20*l*), (10*l*) versus (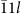)) agree with the anisotropy observed in Fig. 7c, which is also due to the sliding layer effects.

### Model

**Figure 8:**
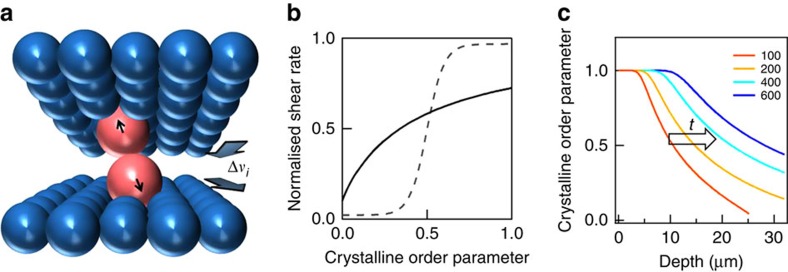
Shear annealing model. (**a**) Schematic ordering forces for individual spheres out of position. (**b**) Nonlinear transfer relation between normalized shear rate and crystalline order in each layer, for polymer opals (solid) and colloids (dashed). (**c**) Resulting crystalline order versus depth for increasing total strain applied (red to blue).

Our data imply that ordering into *hcp* planes proceeds progressively inwards from the flat film surfaces. However, unlike diffusing colloids in solution, which show sharp crystal interfaces^22^, here we find a gradation of ordering. Our comparison of optical and SAXS measurements show that in-plane and stacking-plane order both improve at the same rate (Fig. 5f,g, Supplementary Fig. 25), indicating that they arise from a single complementary process. Since diffusion in this high Peclet number composite is virtually absent, we therefore infer that it is order-dependent shear viscosities that underpin the driving mechanism. As the disordered spheres separate into distinct planes, the shear viscosity drops (Fig. 8a). At the same time, the viscosity also reduces when in-plane ordering leads to neighbouring rows of spheres aligned along the shearing direction. We have previously measured such shear thinning in this system with increasing order^23^,^52^, which although taken on entire films and thus not measuring the local viscosity in each layer discussed here, do however provide a basis for this model. Displaced defect spheres will experience strong restoring forces pushing them into ordered positions (Fig. 8a). This strongly resembles the local focusing of forces in ‘jamming' phenomena^53^. To parameterize this shear thinning, we define a local shear rate 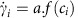 for each layer *i*, which increases as the local crystalline order parameter *c*_*i*_ increases (Fig. 8b). In this lowest order model, *c*_*i*_ is an archetypical direction-averaged order parameter varying from 0 (disordered) to 1 (perfect order), while *a* is proportional to the total shear strain applied across the entire film thickness (see below, Supplementary Discussion). We note that first principles calculations or direct measurements of this transfer function *f*(*c*) are of great interest for quantitative prediction, especially for different sized and shaped nanoparticles.

This allows a simple generic model that captures the crucial elements. Increasing shear strain (as the film bends around each roller) is distributed non-uniformly between different layers depending on their local order *c*_*i*_(*z*_*i*_,*t*) at depth *z*_*i*_ and time *t*. Additional velocity (∝ shear rate) added to each layer generates an interlayer drag force proportional to the velocity difference between neighbouring layers, 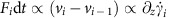, which enhances the order as described above. The resulting improvement of local order in each time step can then be modelled as 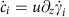 where *u* parametrizes the effectiveness of this shear ordering. Substituting 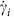 leads to


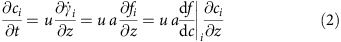


The constant *a*(*t*) corresponds to the shear strain applied and is found by equating each additional shear strain applied across the entire film d*γ*_tot_ with the sum of the distributed non-uniform local additional shear strains in each layer,


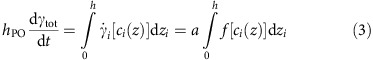


By numerically integrating (1), (2), we obtain spatiotemporal solutions for the development of ordering under applied shear within the sample. Depending on the transfer function *f*(*c*) different behaviours are obtained (Supplementary Discussions). For a sharp threshold between ordered and amorphous regimes (Fig. 8b, dashed), we recover the behaviour of a growth front seen in colloidal systems^22^, with no *ad hoc* assumptions (Supplementary Fig. 38). On the other hand with a more gradual shear thinning as expected here, for instance *f*=(1+*c*_0_/*c*)^−1^ (Fig. 8b, solid), we obtain solutions (Fig. 8c) that strongly resemble those found experimentally (Fig. 6c). In particular, a long-tailed crystallization front flows from the interface, producing a saturated crystalline order behind it (Supplementary Fig. 39).

Despite the simplicity of this model, we find no record of it in previous literature, although it explains many aspects observed. Increasing order in the surface layers reduces their viscosity so that increasing strains are progressively less applied within the bulk interior, being screened out by the easily sheared crystallized surface. Improving deep order thus requires much higher strains, which eventually tears apart crystals in the surface regions. The line defects persisting for U-BIOS restrict shear thinning, and can only be annealed out through line defect annihilation by forces along *y*, which are absent without B-BIOS. This leads to less surface screening with U-BIOS and better ordering in the bulk. On the other hand, B-BIOS provides better surface order, and thus poorer deeper bulk order. Eventually shear ordering fronts from top and bottom surfaces meet in the film centre.

Ordering here relies on irreversible dissipative forces focussed on out-of-place spheres, and is independent of the sign of the velocity difference between neighbouring planes, thus rectifying the shear oscillations. While increasing steady-state shear pulls the crystallites apart, oscillatory shear is able to repeatedly nudge errant nanoparticles towards the lowest viscosity state.

## Discussion

In addition to a clear demonstration of large-scale assembly using this BIOS method, our work provides the first experimental determination of the detailed PO structure, and new physical insights into the microscopic mechanisms behind crystallization of this rheologically unique system. The general principles underpinning BIOS can thus be applied in many ways including ordering of widely varying nanoparticle sizes, and even mixtures with polydispersity exceeding 20% which are normally impossible to order^54^. For instance, anisotropic nanoparticles should have even further improved degrees of ordering, delivering a range of nematic phases in flexible films. This may be particularly interesting in the case of cellulose nanorods, which are known to produce intense structural colours but take days to form chiral nematic phases^55^. The shearing of nanoparticle systems in this fashion also acts to segregate nano components into stacks of ordered two-dimensional layers, opening up many new possible architectures.

## Methods

### Optical measurements

Normal incidence reflection is measured using an Olympus BX51 microscope with a × 5 objective (NA 0.13) coupled to an Ocean Optics QP50 UV-VIS fibre and an Ocean Optics QE65000 spectrometer. The size of the measured spot is ∼20 μm in diameter. Reflection from a silver mirror is taken as the reference. Transmission is measured using collimated incident light from an Ocean Optics DH-2000 UV-VIS-NIR light source coupled via a 400-μm diameter optical fibre and the transmission collected into a second 400-μm diameter optical fibre attached to a QE65000 spectrometer. Transmission of air is taken as a reference.

### FIB-SEM characterization

PO samples for FIB-SEM characterization are first exposed to RuO_4_ vapour for 48 h, and then embedded in dental wax or epoxy resin stubs for cryomicrotoming. After this microtome step, the stubs are coated with a thin layer of platinum and milled by using an FEI 200 FIB system using milling currents from 800 to 200 pA at 30 kV voltage to get clean cross-sections. The stubs are then stained again for another 12 h and coated with an ultra-thin layer of platinum before insertion into a Zeiss Cross-Beam system for further fine milling with 50 to 200 pA currents and SEM imaging at 3 kV voltage. Due to the extreme difficulty and very long times needed for this characterization process, not all samples have been characterized in this way. 3D reconstruction of the nanostructure of the B40 sample is done similarly on an FEI Helios NanoLab SEM-FIB system. Samples are sliced at intervals of ∼20 nm.

### Small-angle X-ray scattering

SAXS measurements are performed on the DUBBLE beamline BM26B at the ESRF in Grenoble using a photon energy of 12.4 keV. The diameter of the beam is ∼300 μm. A Pilatus 1 M 2D CCD detector with pixel size of 22 μm^2^ is used to collect diffraction data, and the detector is placed 7 m from the sample. Background scans are taken every 30 min by measuring scattering in air without samples. The exposure time for each measurement is 60 s. The *q*-range is calibrated using fiber diffraction of wet rat tail collagen. Diffraction data has been combined and processed in custom procedures in Igor (Wavemetrics). The background is subtracted and the intensity of each scattering pattern is normalized using *I*_normalized_=(*I*_sample+air_−*rI*_air_)/*P*_1,sample+air_ in which *I*_normalized_ is the final normalized data after subtracting the background, *I*_sample+air_ is the original diffraction data of the samples, *I*_air_ is the original data of the background, *P*_1_ and *P*_2_ are the values of the upstream ion chamber and downstream ion chamber respectively, and *r*=*P*_2,sample+air_/*P*_2,air_.

### Rheological and mechanical property measurements

Frequency sweep, strain sweep and time sweep tests are performed with an Ares-G2 Rheometer (strain controlled, parallel plate; TA Instruments)at the same temperature of 100 °C on both pure PEA material and green PO (*D*_core−shell_=223 nm) disks of standard test specimen size. Frequency sweep tests are performed with 10% strain for PEA, and 1% for PO since PO is much stiffer than pure PEA. Strain sweep tests for PEA are performed with a frequency of 10 rad s^−1^ and with up to 100% strain, whereas for PO samples it is performed with the same frequency of 10 rad s^−1^ but only up to ∼40% to avoid overload errors. Time sweep tests for both PEA and PO take 1,200 s, with 150% strain for PEA and 50% for PO. Tensile tests of PET foils, PO ribbons (the disordered direct product of extrusion) and PO films are performed on a DMA Q800 instrument. Tensile tests of both PET foil and PO ribbons are performed at both room temperature (20 °C) and 120 °C. Tensile tests of PO films with the same thickness of ∼80 μm before BIOS ordering and after 10 passes of U-BIOS are carried out at room temperature.

### Abaqus simulation

Simulations for bending-induced deformation are performed with the finite element analysis software Abaqus (see http://www.3ds.com/products-services/simulia/products/abaqus/ for details). The model is shown in Supplementary Fig. 4. For both PET and PO layers, CPS4R elements are used with reduced integration and hourglass control. For the remaining part of the model, CPS3 elements are used. An orthogonal material orientation is assigned to both PET and PO layers with the *x* direction parallel to the surface and *z* direction as the thickness direction (*x* and *z* are equivalent to the built-in 1 and 2 coordinates in the software). The isotropic elastic model is used for PET with a Poisson ratio of 0.4. A hyperelastic isotropic material model is used for PO. The material is defined by using data from tensile tests. A series of different ratios between the Young's moduli of PET and PO are used to evaluate the effects of relative rigidity. Frictionless tangential behaviour is used for the contact, with hard contact for normal behaviour. The Nlgeom option, accounting for geometric nonlinearity, is switched on. For shear thinning simulations, the material properties of the top and bottom quarter depth of the PO layer are set at half the Young's modulus of the middle of the PO. The rest of the settings of the simulation remain unchanged.

### Data availability

All relevant data present in this publication can be accessed at: https://www.repository.cam.ac.uk/handle/1810/255824.

## Additional information

**How to cite this article:** Zhao, Q. *et al*. Large-scale ordering of nanoparticles using viscoelastic shear processing. *Nat. Commun.* 7:11661 doi: 10.1038/ncomms11661 (2016).

## Supplementary Material

Supplementary Information Supplementary Figures 1-39 and Supplementary Discussion

Supplementary Movie 1 Abaqus simulation on log scale of normal strain log(Hxx) (LE11) inside the PET-PO-PET sandwich beam in bending, with ratio between Young's moduli of PET and PO of 2000:5.

Supplementary Movie 2 Abaqus simulation on log scale of shear strain log(Hxy) (LE12) inside the PET-PO-PET sandwich beam in bending, with ratio between the Young's moduli of PET and PO of 2000:5.

Supplementary Movie 3 Shear banding phenomenon in Abaqus simulation (LE12). The PO layer is comprised of three layers, the top and bottom layers have only half the Young's modulus of the middle layer.

Supplementary Movie 4 View of the stacking of the hcp planes within a selected cube, imaging is from the depth-y plane. The video is constructed from FIB-SEM experiment results from a sample with 40 passes of B-BIOS.

## Figures and Tables

**Figure 1:**
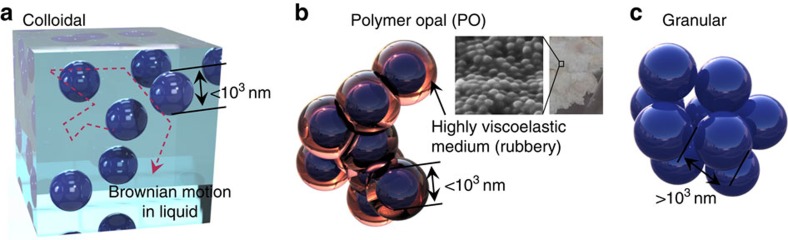
Comparison of polymer opals with colloidal and granular systems. (**a**) Dielectric spheres in water. (**b**) Core-shell nanoparticles in PO stick to each other via the viscoelastic shells (inset shows SEM image of the spheres in the matrix, and nanoparticles dried from emulsion polymerization). (**c**) Granular spheres piled together give bridging networks.
