# Supplementary material for: Large-scale ordering of nanoparticles using viscoelastic shear processing
Source: Nat Commun. 2016 Jun 3;7:11661. doi: 10.1038/ncomms11661 (PMC4895715; doi:10.1038/ncomms11661)
Supplement: Supplementary Information — Supplementary Figures 1-39 and Supplementary Discussion [file ncomms11661-s1.pdf]

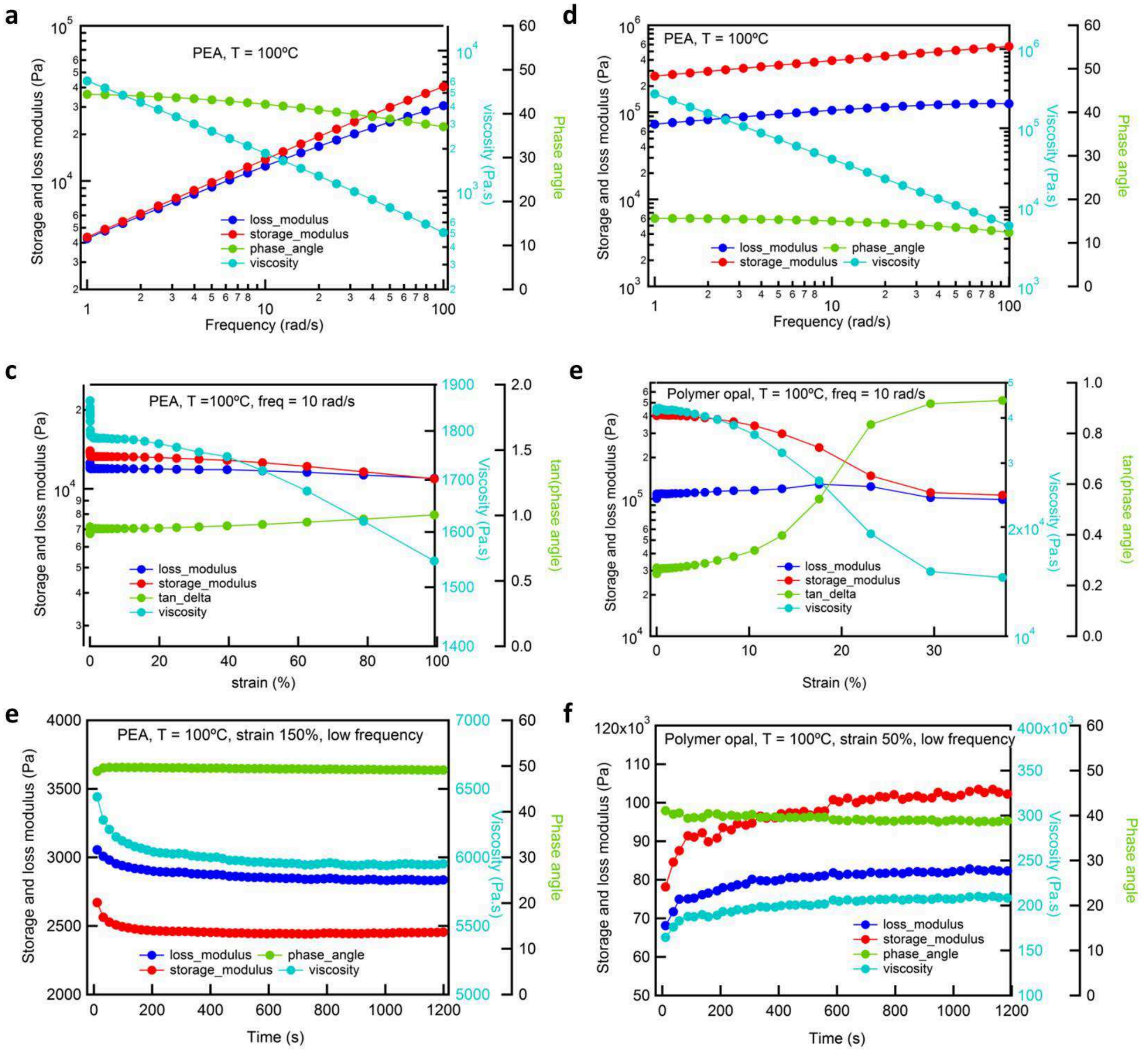

Supplementary Figure 1. Characterisation of the viscoelastic properties of PEA and POs by an Ares Rheometer. **a**, Frequency sweep test of PEA at 100 °C. **b**, Frequency sweep test of PO at 100 °C. **c**, Strain sweep test of PEA at 100 °C and a frequency of 10 rad/s. **d**, Strain sweep test of PO at 100 °C and a frequency of 10 rad/s. **e**, Time sweep test of PEA at 100 °C, 150% strain, and low frequency. **f**, Time sweep test of PO at 100 °C, 50% strain, and low frequency.

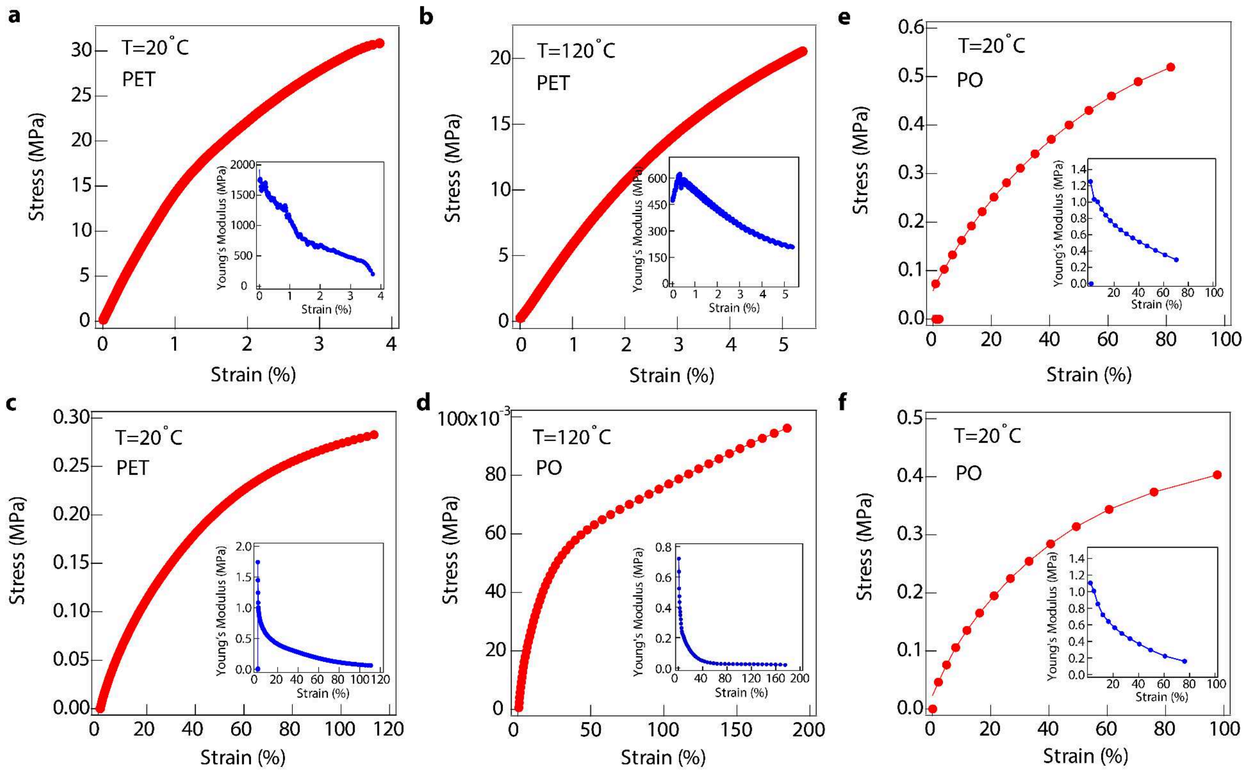

Supplementary Figure 2. Tensile tests of PET and PO films at different temperatures using a DMA Q800. **a**, Stress-strain curve of PET foil at T=20 °C, inset, calculated Young's modulus. **b**, Stress-strain curve of PET foil at T=120 °C, inset, calculated Young's modulus. **c**, Stress-strain curve of PO after extrusion at T=20 °C, inset, calculated Young's modulus. **d**, Stress-strain curve of PO at T=120 °C after extrusion, inset, calculated Young's modulus. **e**, Stress-strain curve of PO at T=20 °C after rolling-lamination, inset, calculated Young's modulus. **f**, Stress-strain curve of PO at T=20 °C after 10 passes of U-BIOS process, inset, calculated Young's modulus.

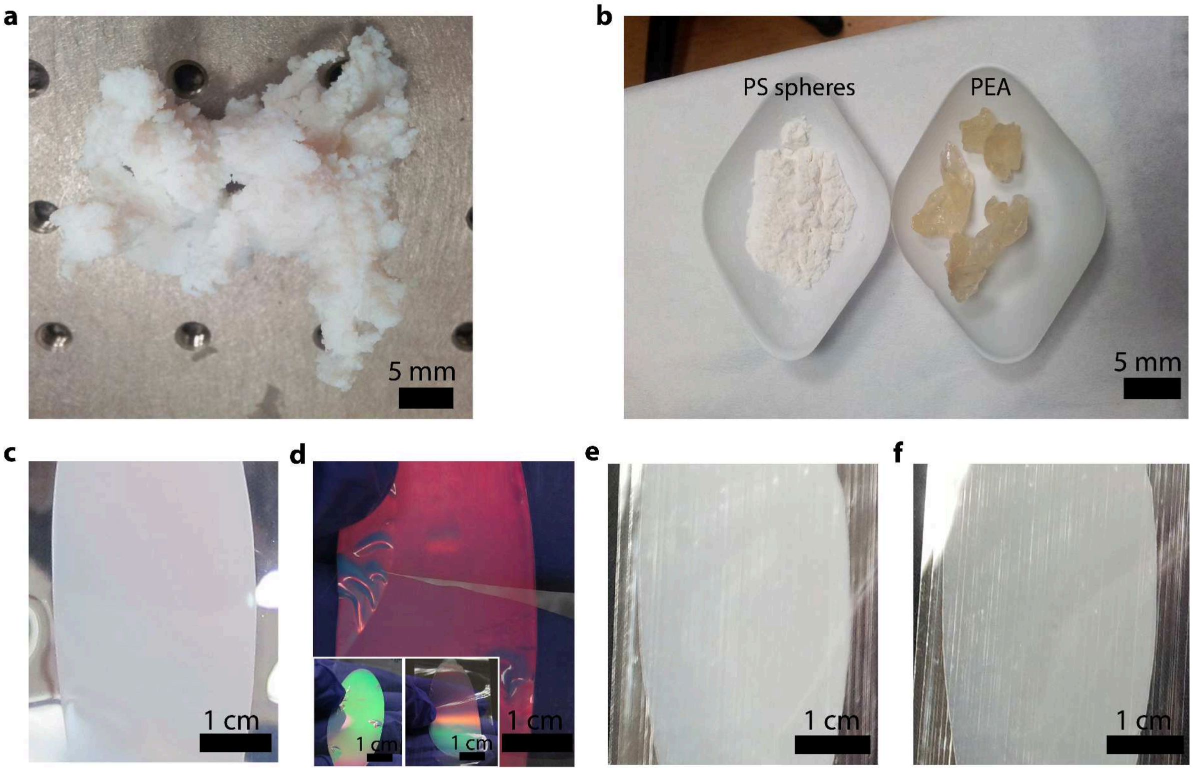

Supplementary Figure 3. Difference between POs fabricated from PS-PMMA-PEA core-shell spheres and from a mixture of PS beads with PEA. **a**, Raw materials comprised of core-shell spheres, diameter of each core-shell sphere is $\sim$ 260 nm, with $\sim$ 220 nm PS core. **b**, PS beads (left) and PEA (right) before mixing, PS beads are the same size as the PS core in the core-shell spheres. **c**, PO film comprised of core-shell spheres fabricated by standard PO fabrication process before applying BIOS, normal view. **d**, The same sample after 20 passes of U-BIOS. Inset left, tilted (30°) view. Inset right, view under bending and tilting. **e**, Sample fabricated with PS beads and PEA through the same procedure before applying BIOS. **f**, Sample in **e** after 20 passes of U-BIOS.

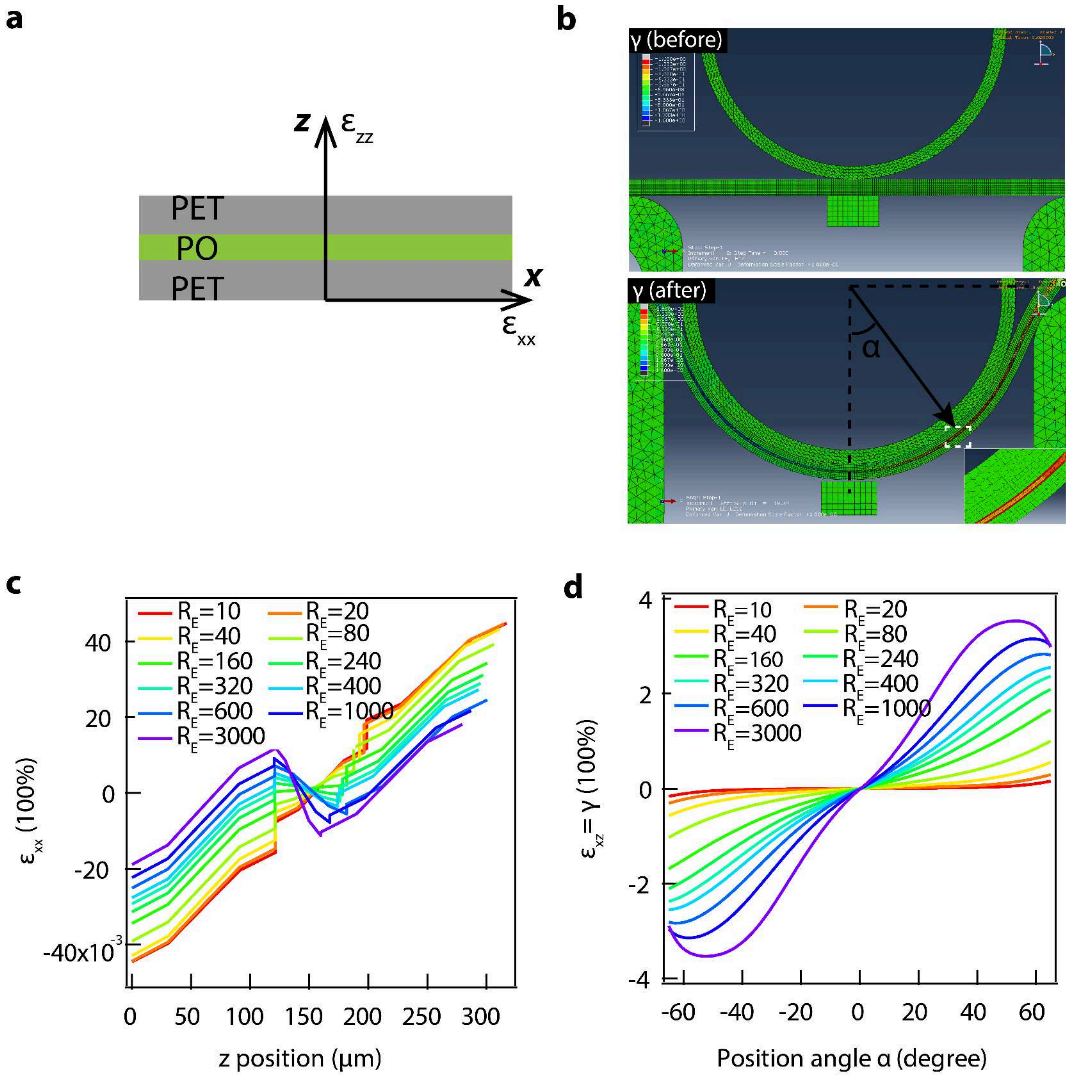

Supplementary Figure 4. Abaqus simulation for shear strain (γ) in bending the PET-PO-PET composite beam. **a**, Schematic view of the 2D sandwich beam model and coordinates, ε_{xx} (ε_{zz}) are normal strains along the longitudinal (thickness) directions, ε_{xz} is shear strain γ. **b**, Simulation snapshots. Top: initial state of model, with beam placed between two holders on either side and rounded tip indenter just touching beam in the middle (block below beam moves with indenter but not in contact so can be neglected). Bottom: shear strain distribution when beam is bent around indenter, with shear mainly in PO layer. Inset, shear banding occurs when PO structures next to boundaries are less resistant to shear. **c**, Normal strain ε_{xx} with depth, and **d**, Shear strain ε_{xz} inside PO with angle ϕ, both for different ratios (R_e) of Young's modulus of PET to PO film.

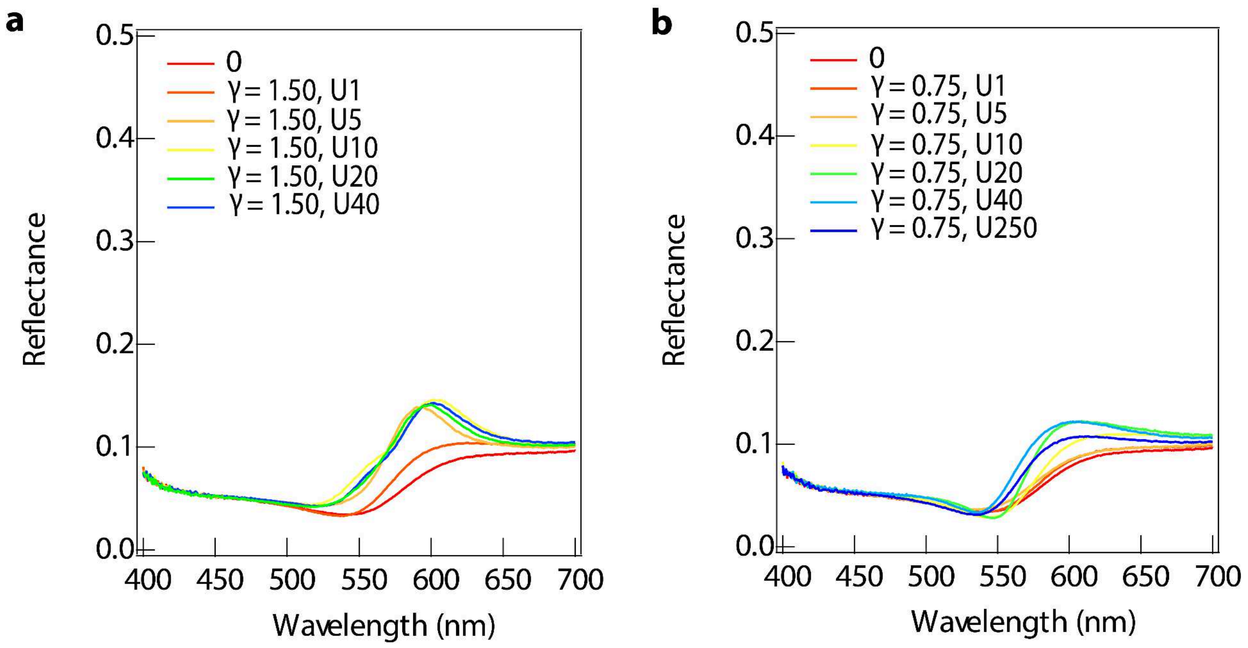

Supplementary Figure 5. Optical properties of PO samples fabricated at 100 °C with U-BIOS for different shear strains. **a-b**, Reflectance spectra of samples ordered with different number of shear oscillations under 150% and 75% strain respectively.

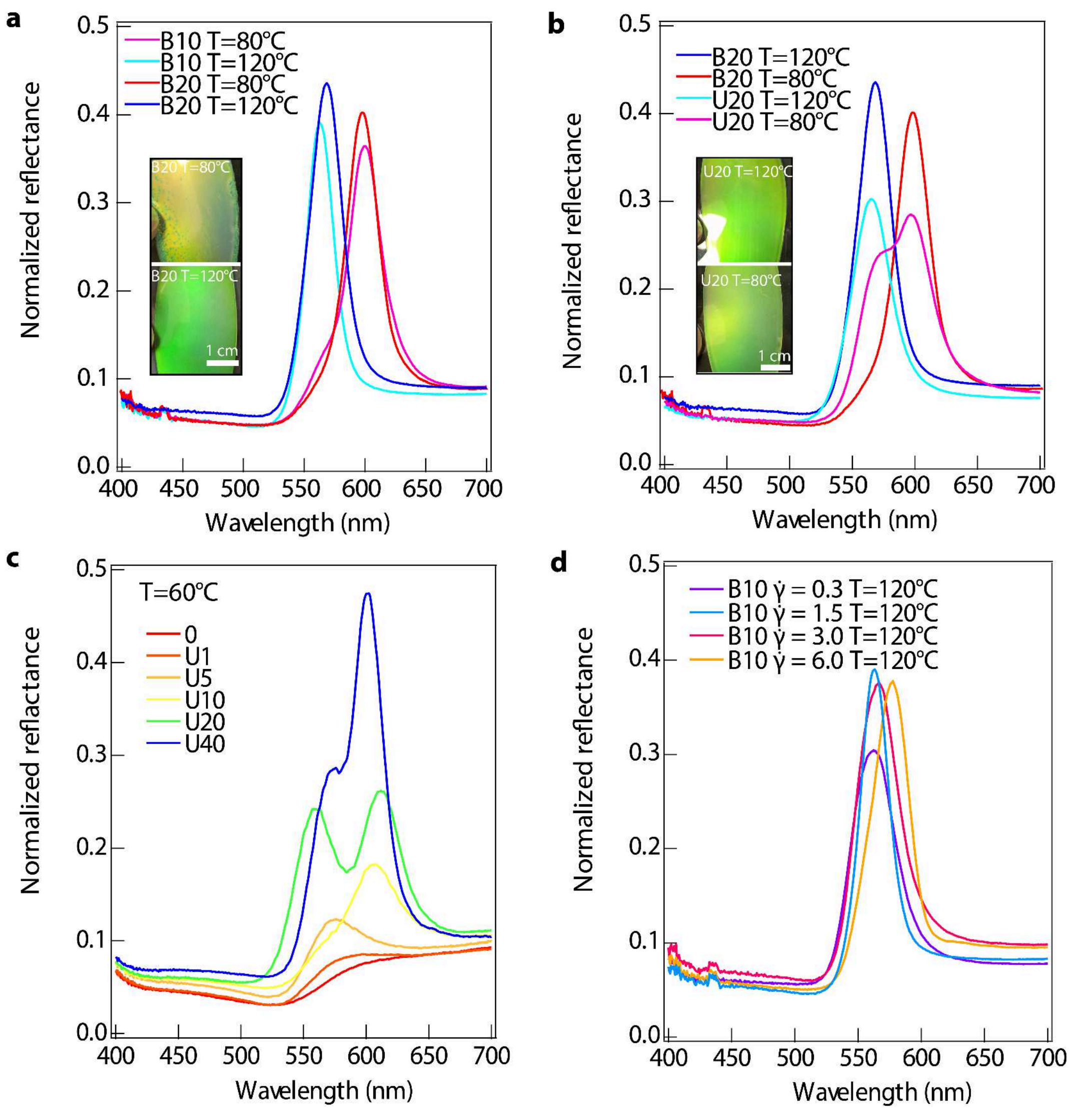

Supplementary Figure 6. Optical properties of POs fabricated with different BIOS methods, temperatures, and shear rates ($\dot{\gamma}$). **a**, Reflectance of samples after 10 and 20 passes of B-BIOS at 80 °C and 120 °C respectively. **b**, Reflectance of samples after 20 passes of B-BIOS and U-BIOS at 80 °C and 120 °C respectively. Insets show visual appearance of these different samples at normal incidence. **c**, Reflectance of samples after different amount of U-BIOS at 60 °C. **d**, Reflectance spectra of samples after 10 passes of B-BIOS at 120 °C, 300% shear strain, but different shear rates ($\dot{\gamma}$). All spectra are obtained at normal incidence.

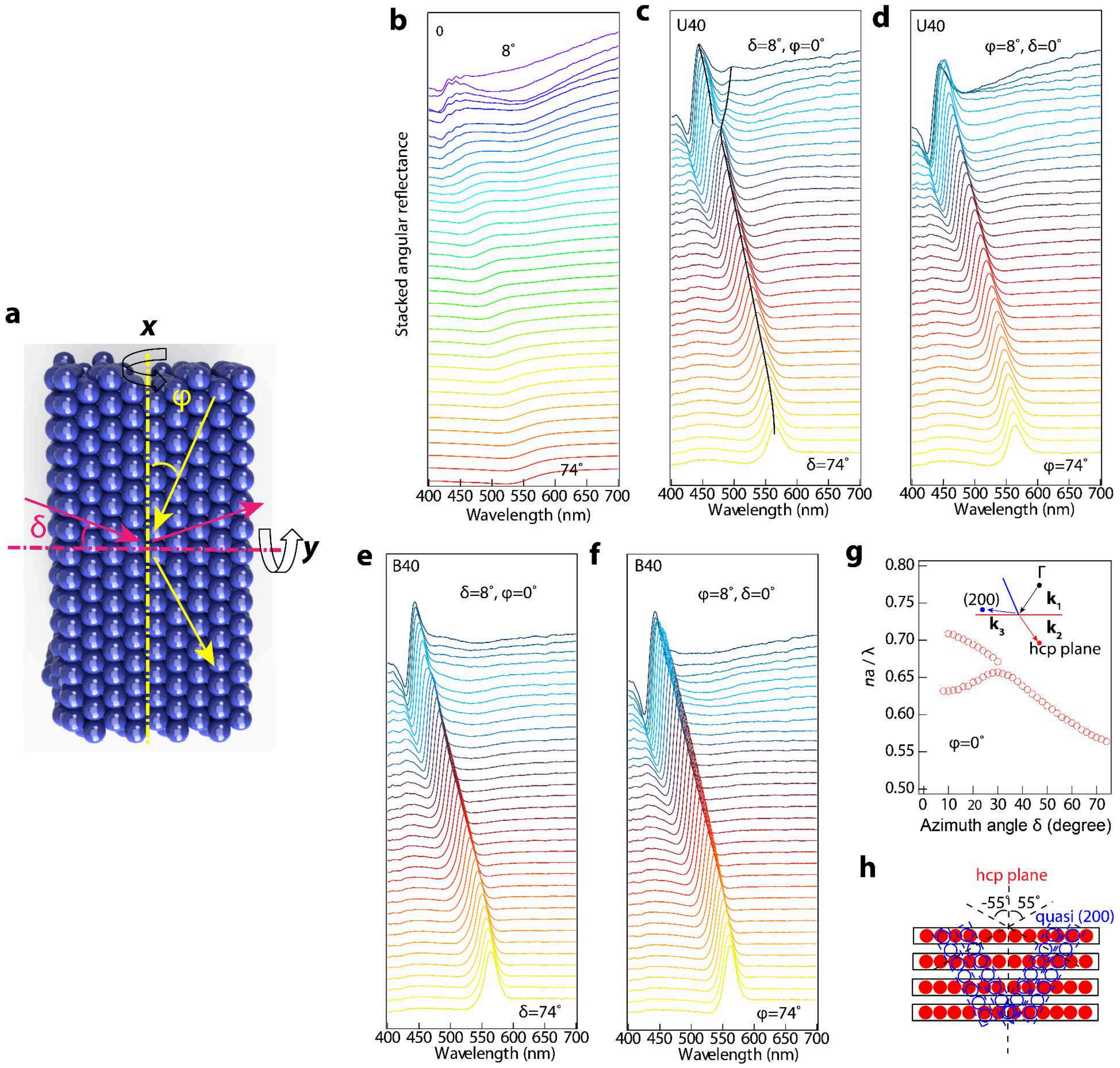

Supplementary Figure 7. Angular dependent reflection of PO samples. **a**, Measurement geometry, δ is the azimuth angle, φ is the elevation angle. **b**, Reflectance spectra of PO before BIOS. **c**, Reflectance of sample U40 with varying δ. **d**, Reflectance of sample U40 with varying φ. **e**, Reflectance of sample B40 with varying δ. **f**, Reflectance of sample B40 with varying φ. **g**, Reflection peaks vs angle (in frequency) from **c**, $\bar{n}$ is mean refractive index, a is nearest sphere distance, λ is wavelength. Inset shows Brilloin zone of PO, $\boldsymbol{k_1}$ is incident wave vector, $\boldsymbol{k_2}$ is diffracted wave vector relative to *hcp* planes, $\boldsymbol{k_3}$ is diffracted wave vector relative to quasi-(200) planes. **h**, Illustration of quasi-(200) planes (blue) with respect to surface *hcp* planes (red).

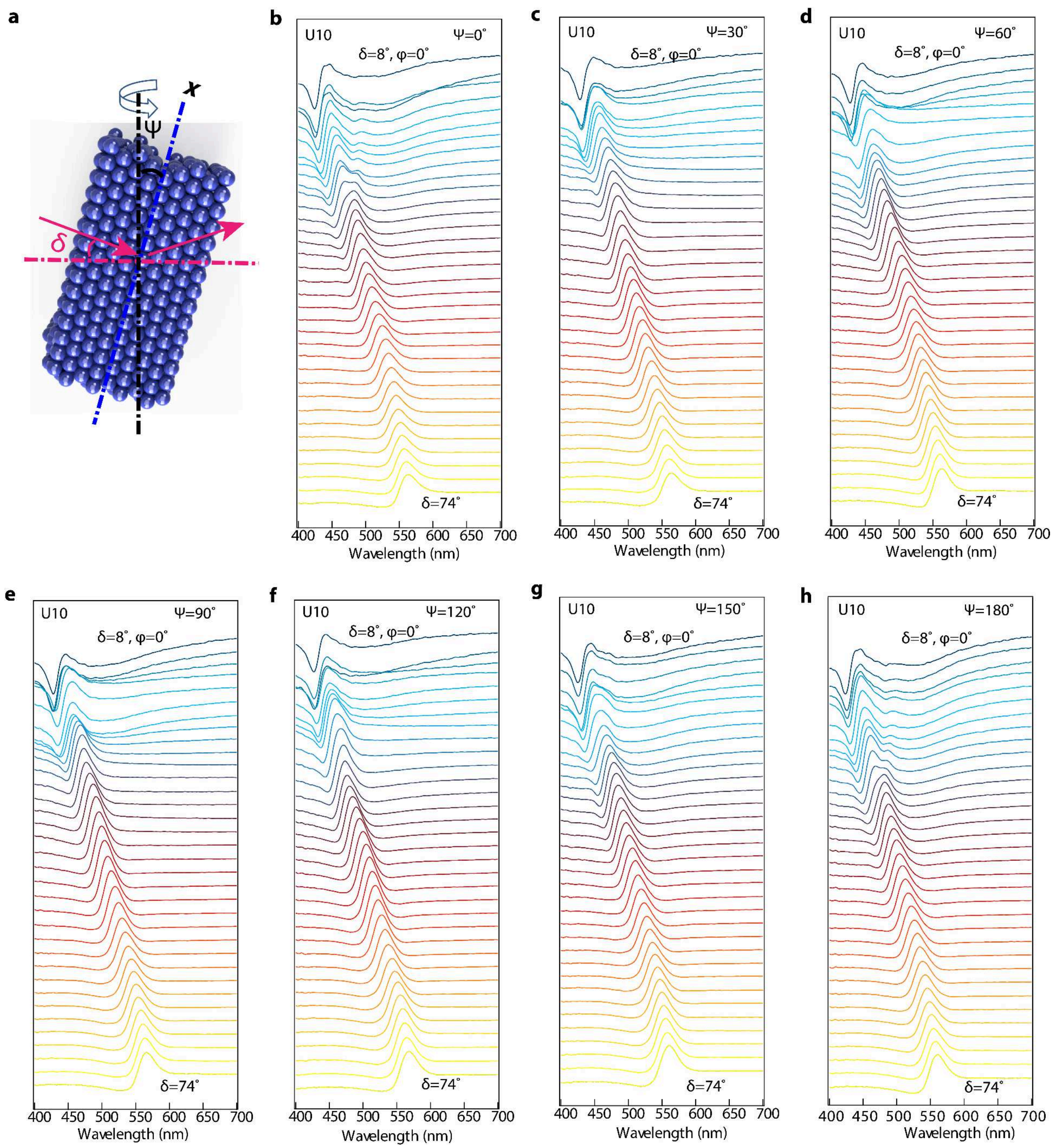

Supplementary Figure 8. Angular dependent oriented reflection of PO films after 10 passes of U-BIOS. **a**, Measurement geometry with x axis rotated off vertical direction by angle Ψ (orientation angle), δ is incident angle from horizontal (azimuthal angle), sample rotated around vertical axis (black dashed line) at each Ψ. **b-h**, Scanning δ for **b** $\Psi = 0°$, **c** $\Psi = 30°$, **d** $\Psi = 60°$, **e** $\Psi = 90°$, **f** $\Psi = 120°$, **g** $\Psi = 150°$, and **h** $\Psi = 180°$. In all cases $\varphi = 0°$.

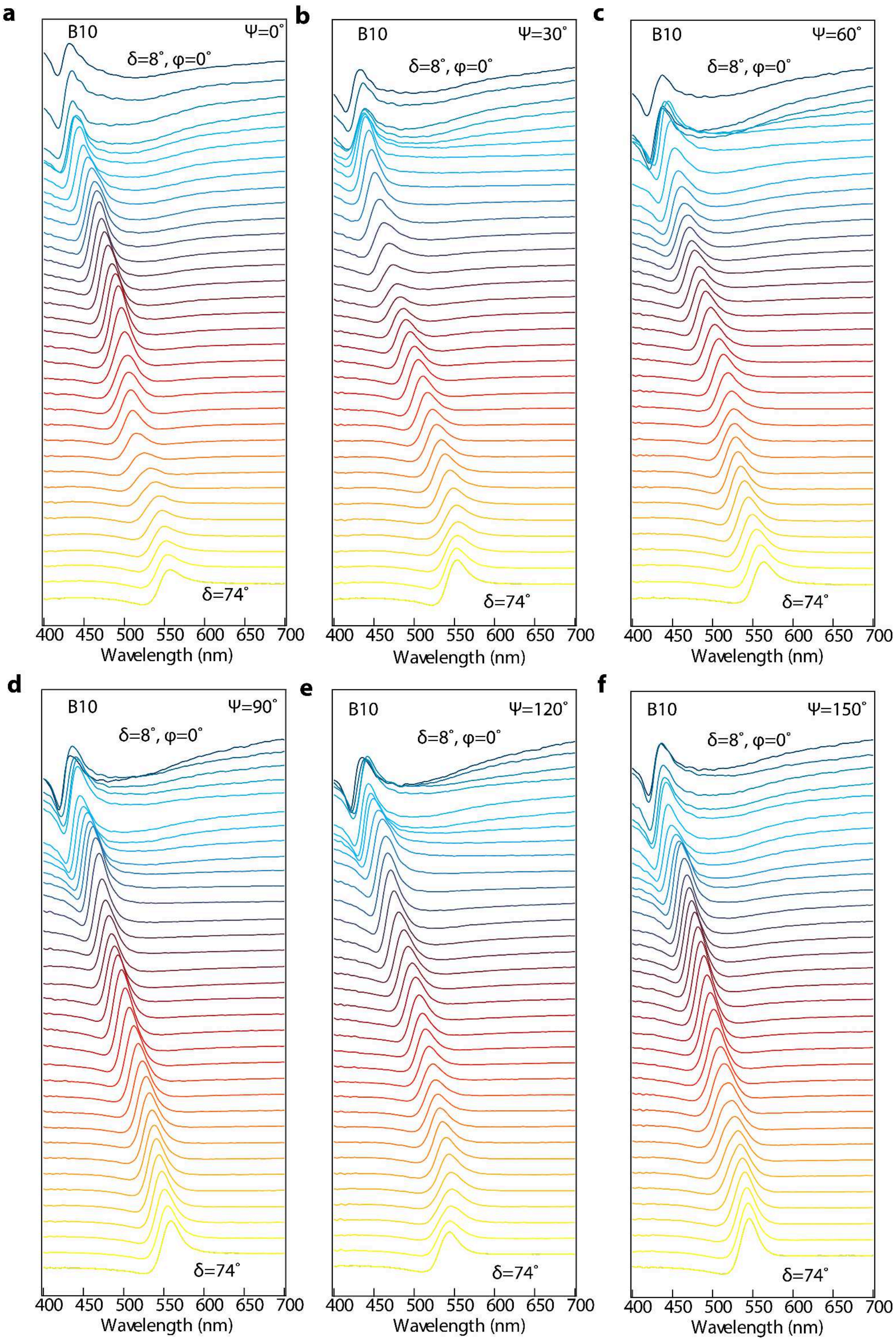

Supplementary Figure 9. Angular dependent oriented reflection of PO films after 10 passes of B-BIOS, measurement geometry as in Figure S11. **a-f**, Scanning δ for **a** $\Psi = 0°$, **b** $\Psi = 30°$, **c** $\Psi = 60°$, **d** $\Psi = 90°$, **e** $\Psi = 120°$, and **f** $\Psi = 150°$. In all cases $\varphi = 0°$.

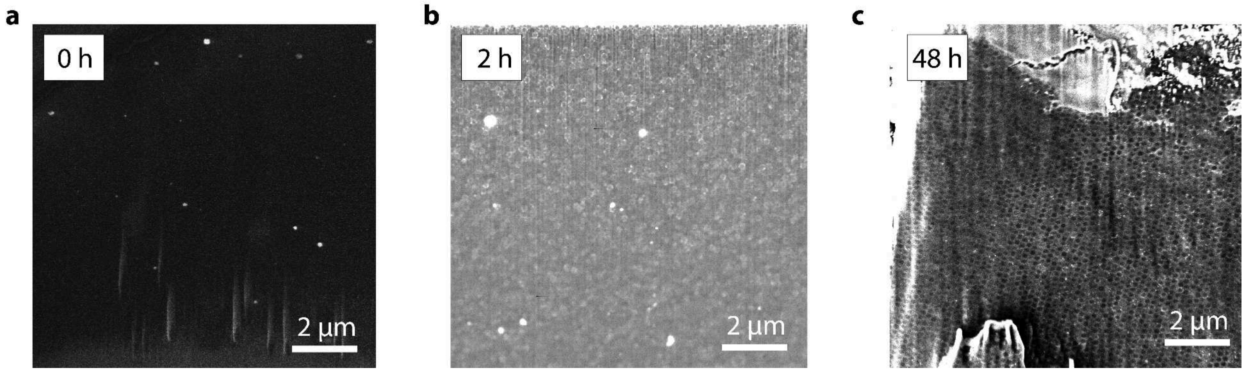

Supplementary Figure 10. Effect of RuO_4 staining on the SEM imaging quality for POs. **a**, SEM image of sample cross-section after FIB milling without staining. **b**, Staining the sample with RuO_4 for 2 hours. **c**, After 48 hours of staining, dark spheres seen in image are the PS cores.

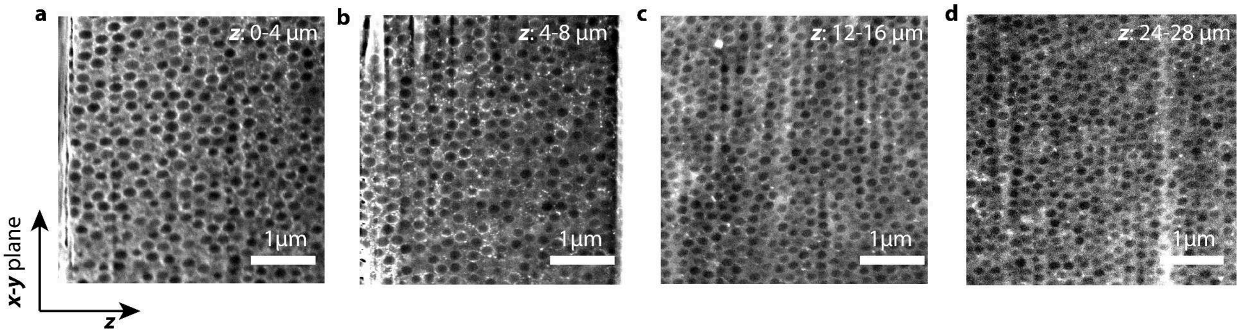

Supplementary Figure 11. SEM cross-sections of PO samples without BIOS, trimmed by FIB. **a**, Cross-section from sample surface (left edge) to depth of $\sim$ 4 μm (right edge), **b**, from 4-8 μm, **c**, from 12-16 μm, and **d** from 24-28 μm.

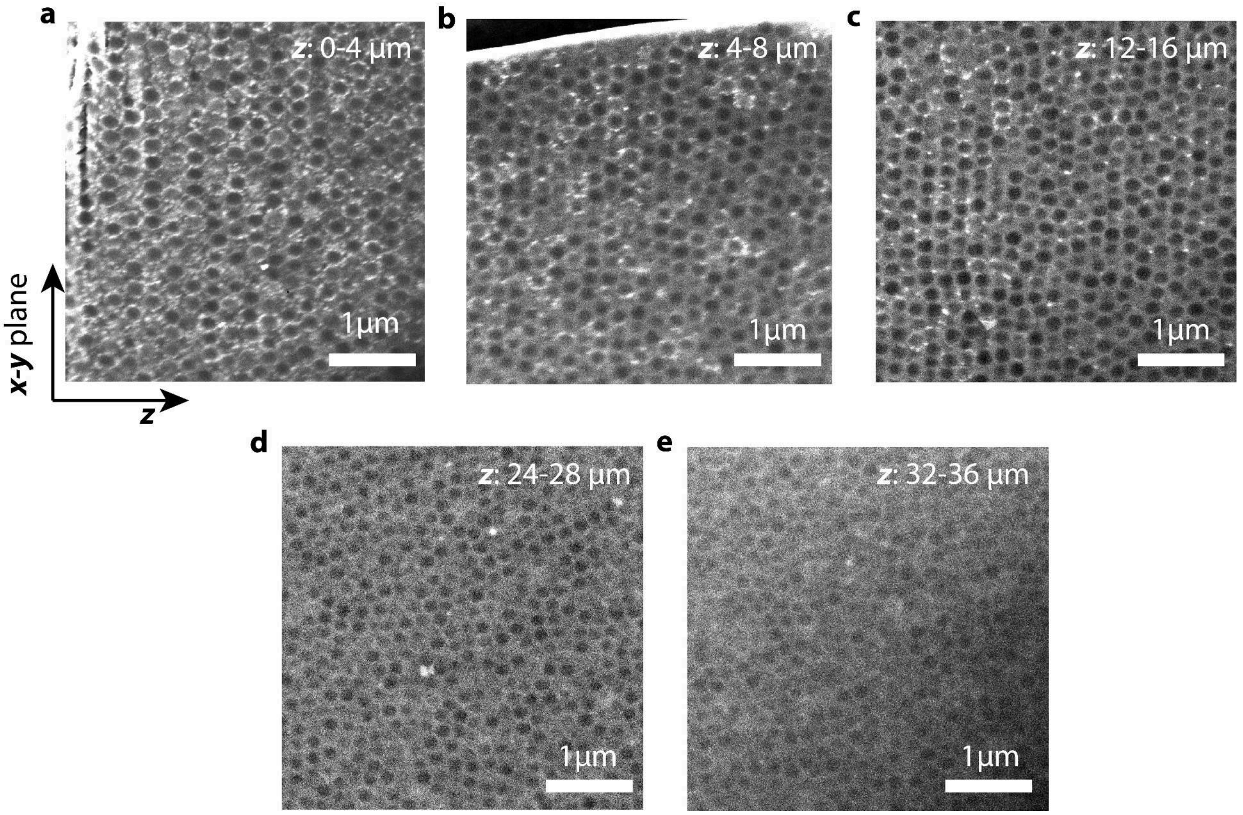

Supplementary Figure 12. SEM cross-sections of PO sample with 1 pass of B-BIOS. **a**, Cross-section from sample surface (left edge) to depth of $\sim$ 4 μm (right edge), **b** from 4-8 μm, **c**, from 12-16 μm, **d**, from 24-28 μm, and **e**, from 32-36 μm.

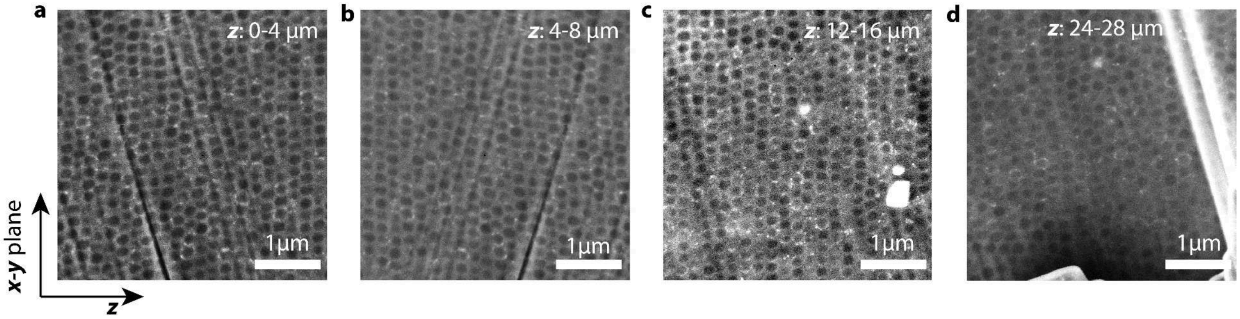

Supplementary Figure 13. SEM cross-sections of PO sample with 5 passes of B-BIOS. **a**, Cross-section from sample surface (left edge) to depth of $\sim$ 4 µm (right edge), **b**, from 4-8μm, **c**, from 12-16 µm, and **d**, from 24-28 µm.

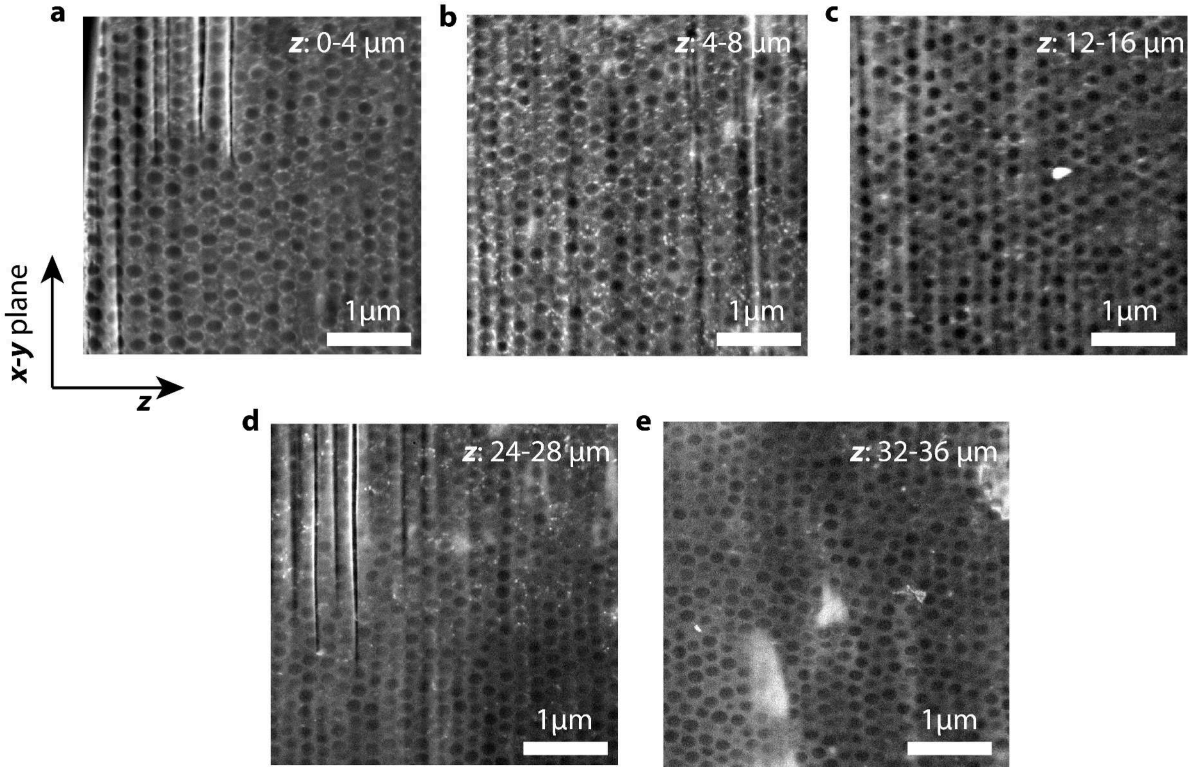

Supplementary Figure 14. SEM cross-sections of PO sample with 10 passes of B-BIOS. **a**, Cross-section from sample surface (left edge) to depth of $\sim$ 4 µm (right edge), **b**, from 4-8 µm, **c**, from 12-16 µm, **d**, from 24-28 µm and **e**, from 32-36 µm.

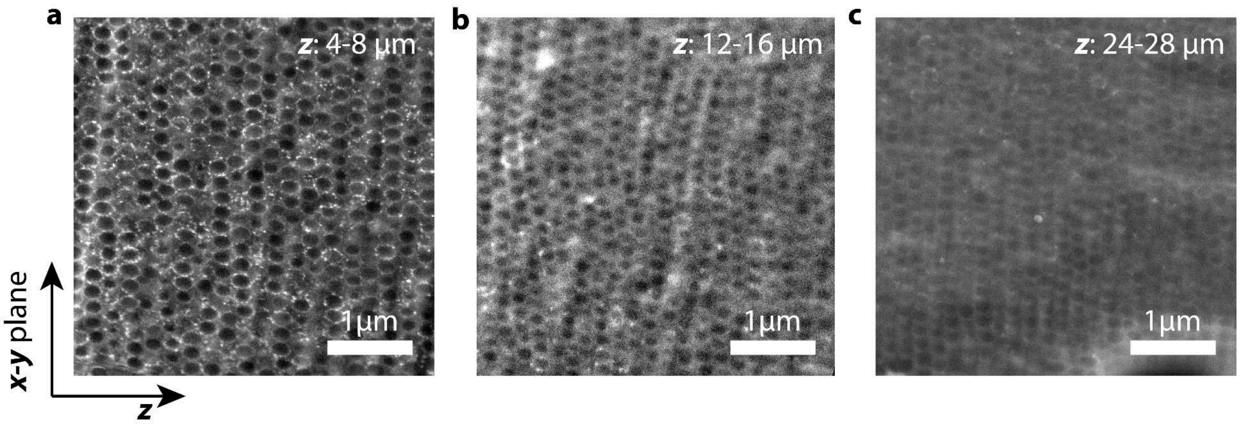

Supplementary Figure 15. SEM cross-sections of PO sample with 20 passes of B-BIOS. **a**, Cross-section from depths of 4-8 μm, **b**, from 12-16 μm, **c**, from 24-28 μm.

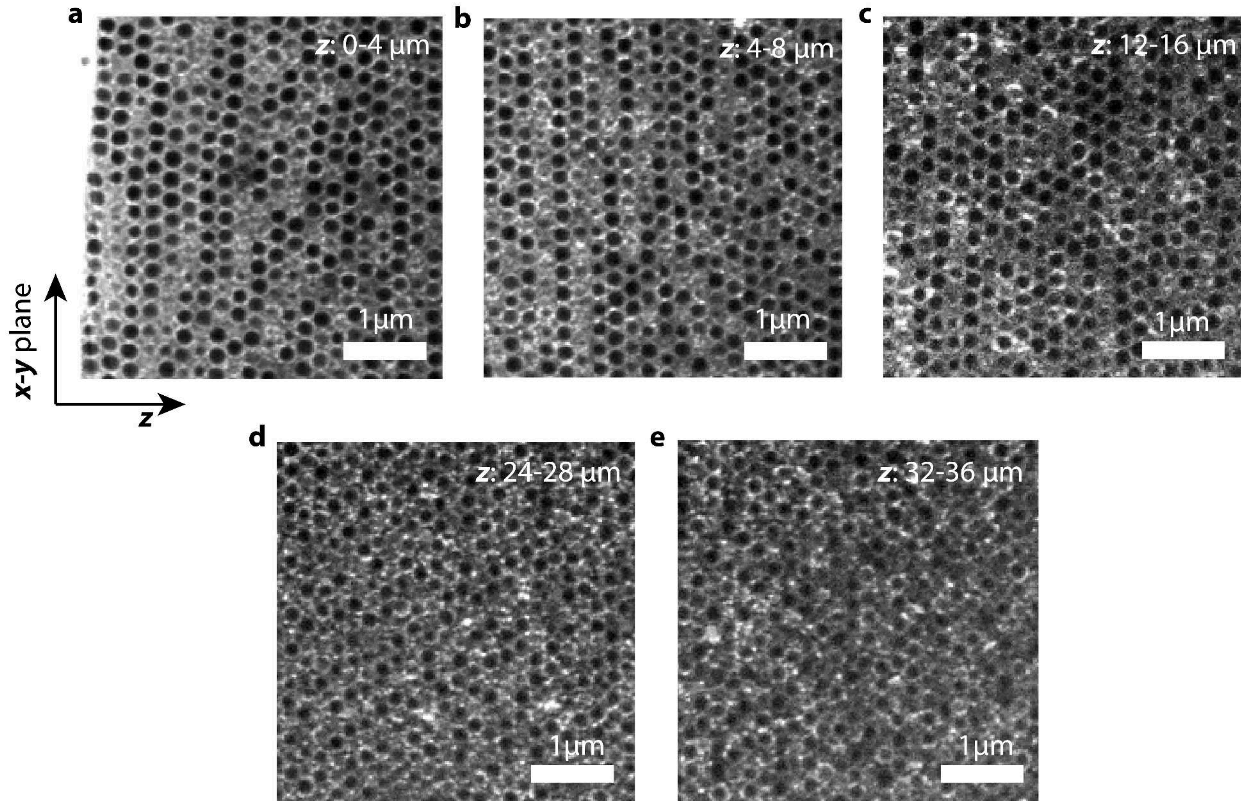

Supplementary Figure 16. SEM cross-sections of PO sample with 40 passes of B-BIOS. **a**, Cross-section from sample surface (left edge) to depth of $\sim$ 4 μm (right edge), **b**, from 4-8 μm, **c**, from 12-16 μm, **d**, from 24-28 μm, and **e**, from 32-36 μm.

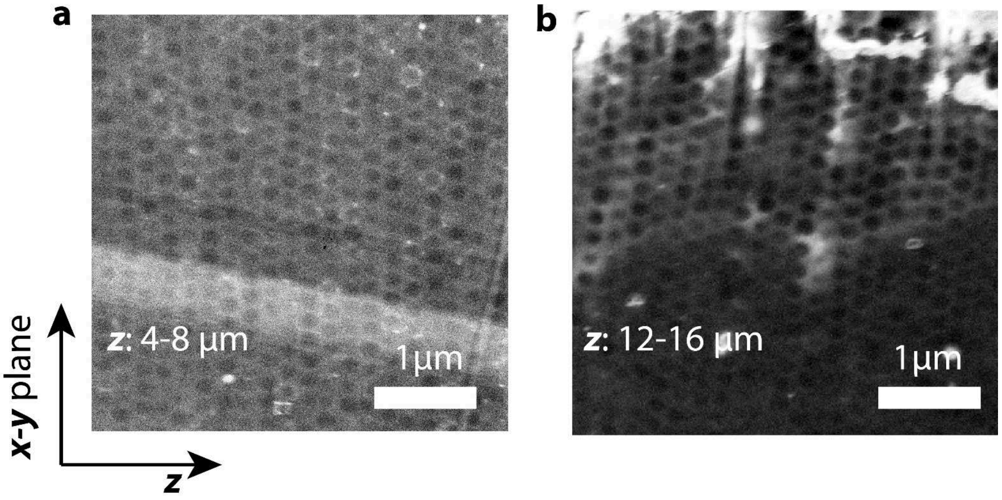

Supplementary Figure 17. SEM cross-sections of PO sample with 10 passes of U-BIOS. **a**, Cross-section from sample surface (left edge) to depth of $\sim$ 4 μm (right edge), and **b**, from 4-8 μm.

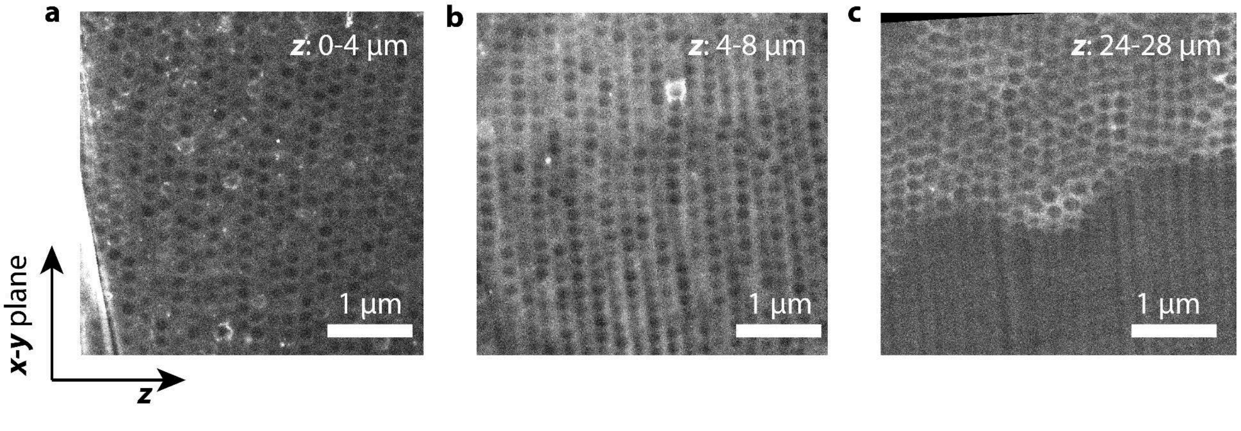

Supplementary Figure 18. SEM cross-sections of PO sample with 20 passes of U-BIOS. **a**, Cross-section from sample surface (left edge) to depth of $\sim$ 4 μm (right edge), **b**, from 4-8 μm, **c**, from 24-28 μm.

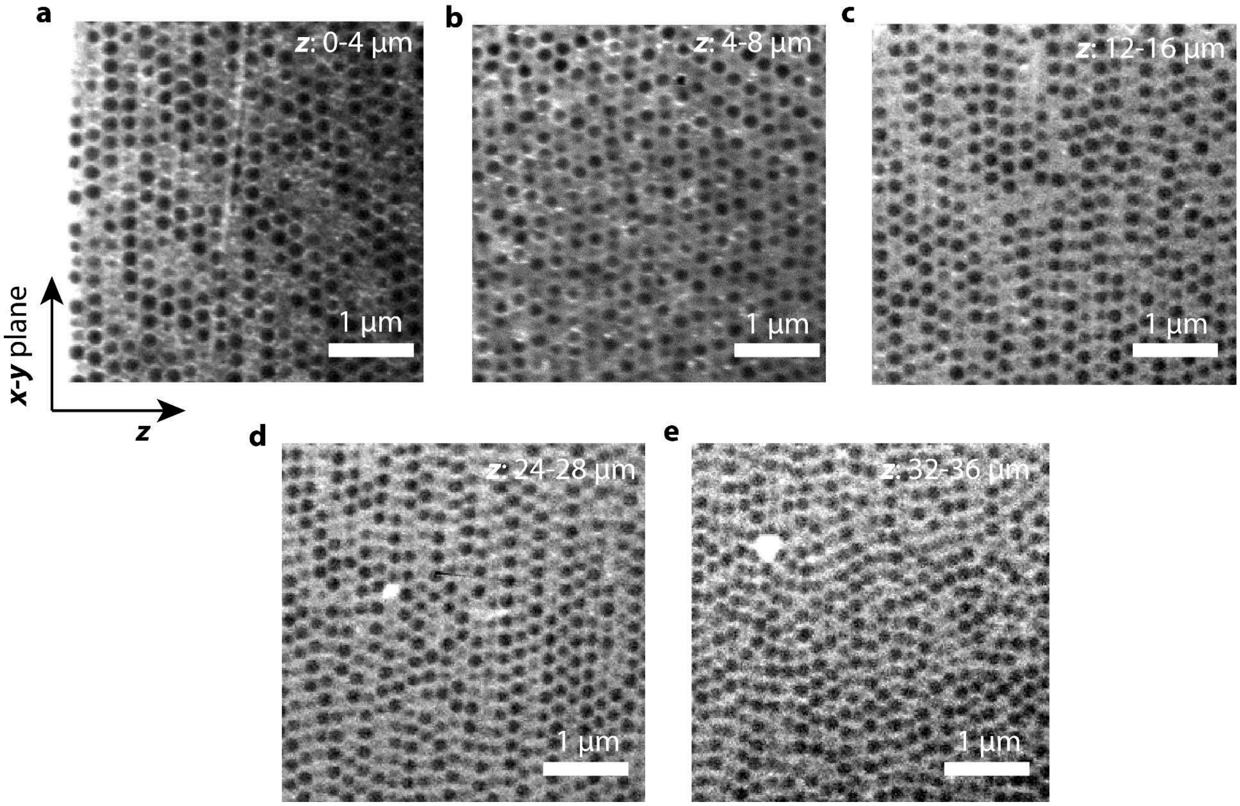

Supplementary Figure 19. SEM cross-sections of PO sample with 40 passes of U-BIOS. **a**, Cross-section from sample surface (left edge) to depth of $\sim$ 4 µm (right edge), **b**, from 4-8 µm, **c**, from 12-16 µm, **d**, from 24-28 µmand **e**, from 32-36 µm.

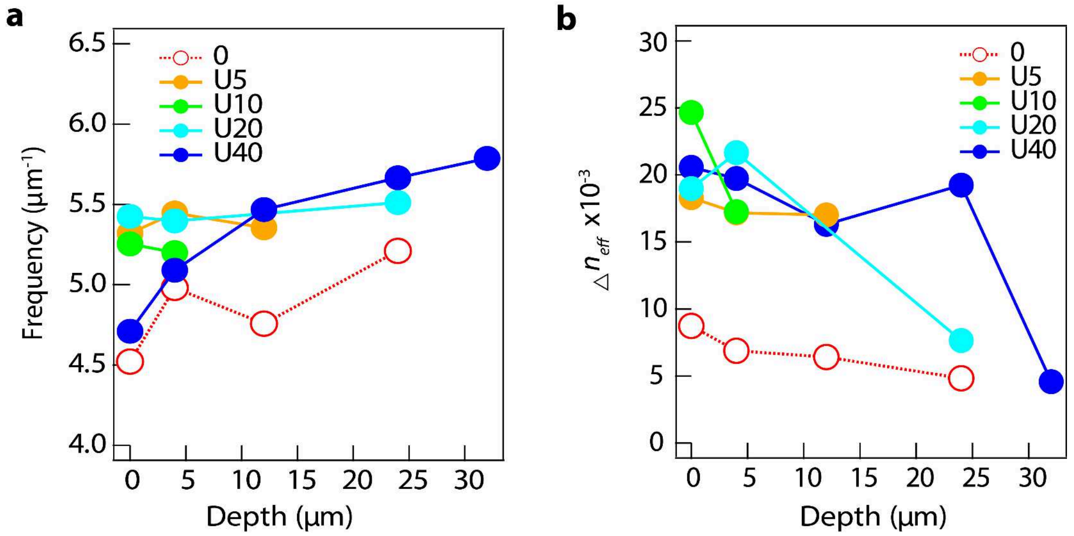

Supplementary Figure 20. The spatial frequency (F) and effective refractive index contrast (Δn_{eff}) of the layered structures in the depth direction of U-BIOS samples. **a**, Change of the spatial frequency. **b**, Change of the Δn_{eff}.

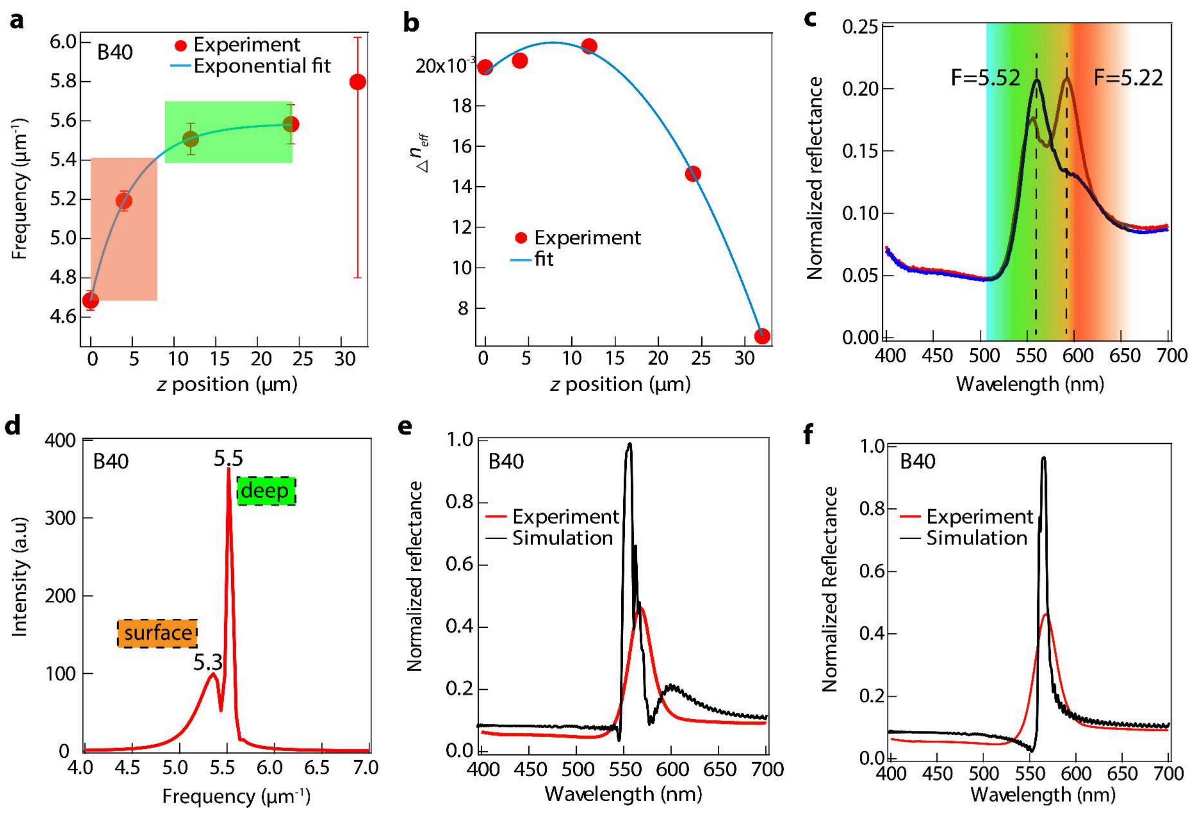

Supplementary Figure 21. Structural analysis extracted from FIB-SEM results of B40 PO sample. **a**, Experimental spatial frequency of *hcp* planes at different depths. **b**, Change of Δn_{eff} of sample at different depths. **c**, Reflectance of two samples both fabricated with 20 passes of U-BIOS at room temperature. Double-peak features seen in both samples with green peak ($F \approx 5.52$) and red peak ($F \approx 5.22$). **d**, FFT of the spatial frequency distribution from **a**. **e**, Measured reflectance of sample and simulation results from transfer matrix model. **f**, Experimental and simulation results from a corrected model, with frequency of deeper part (green region in **a**) reduced by $\Delta F = 0.08$.

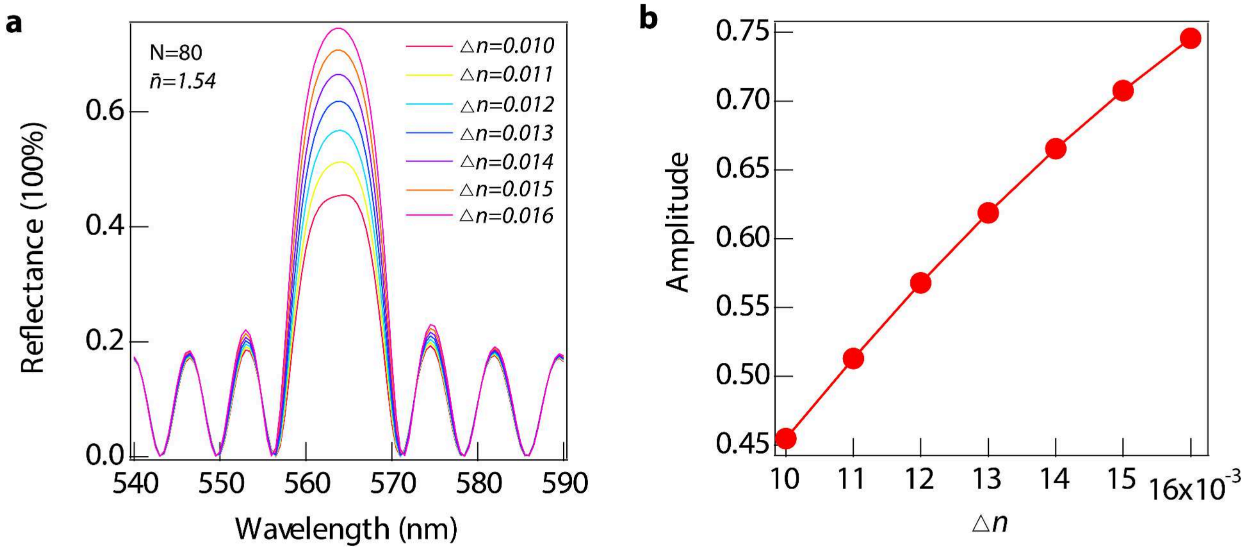

Supplementary Figure 22. Calculated reflectance spectra from model with different effective refractive index contrast Δn values. **a**, Model spectra calculated from 80 layers with mean refractive index $\bar{n} = 1.54$. **b**, Extracted peak amplitude of spectra vs Δn, showing near linear dependence up to R=0.7.

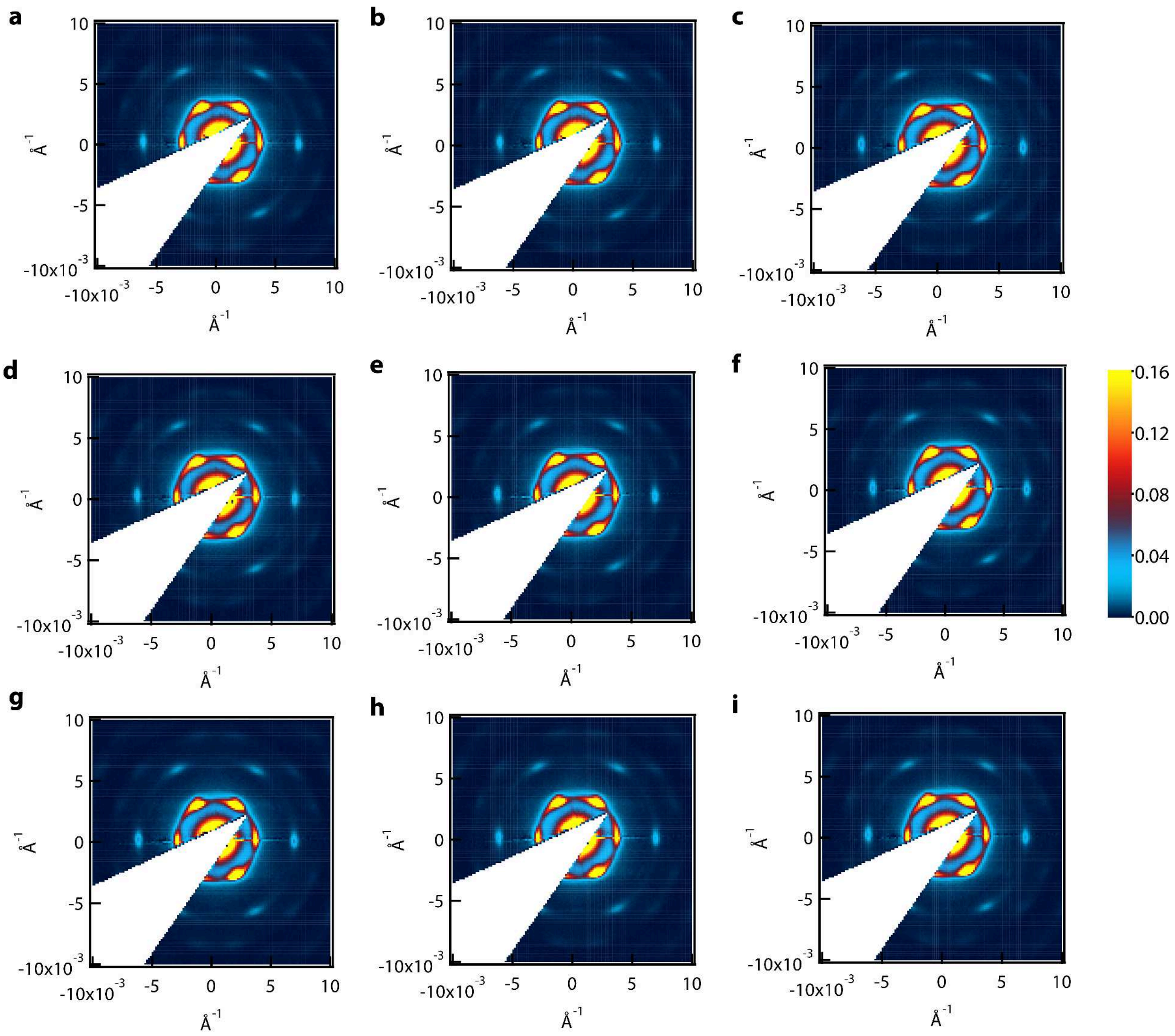

Supplementary Figure 23. SAXS characterisation of the homogeneity of the microstructure in the PO sample after 40 passes of B-BIOS over a 3 mm × 3 mm square area. **a-i**, SAXS patterns from the sample recorded at coordinate positions (in mm) of **a** (-1.5, 1.5), **b** (0, 1.5), **c** (1.5, 1.5), **d** (-1.5, 0), **e** (0, 0), **f** (1.5, 0), **g** (-1.5, -1.5), **h** (0, -1.5), and **i** (-1.5, -1.5).

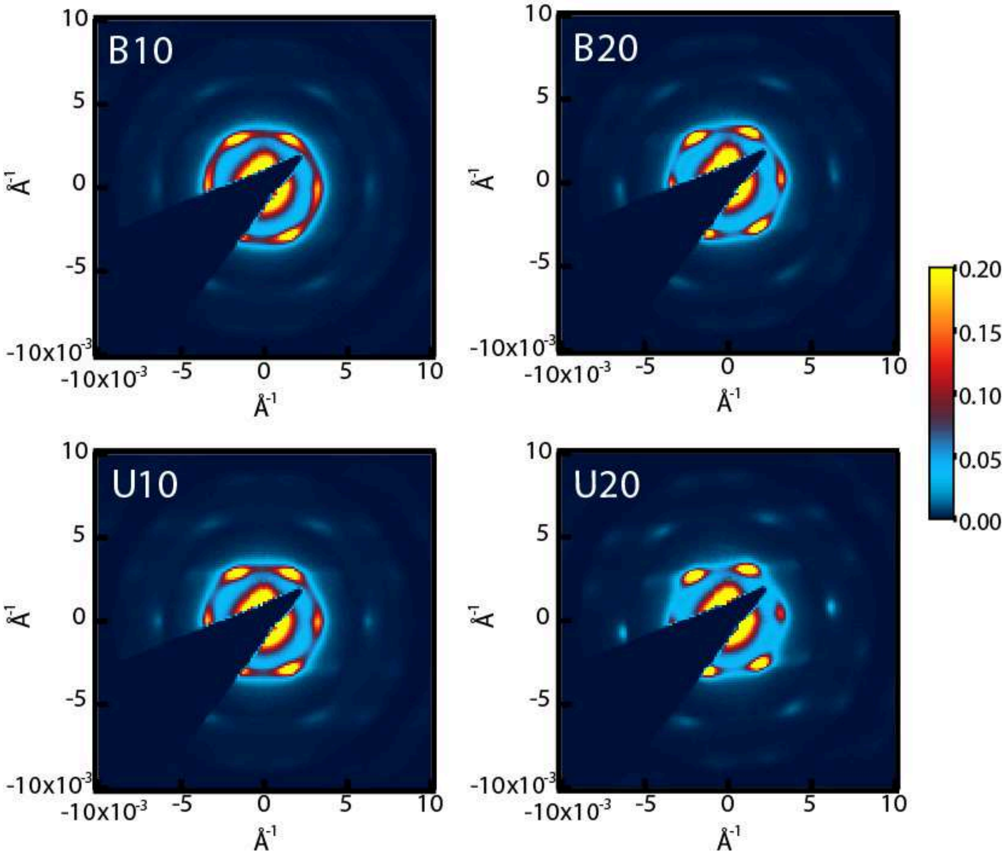

Supplementary Figure 24. SAXS patterns of PO samples after 10 and 20 passes of U-BIOS and B-BIOS respectively at normal incidence.

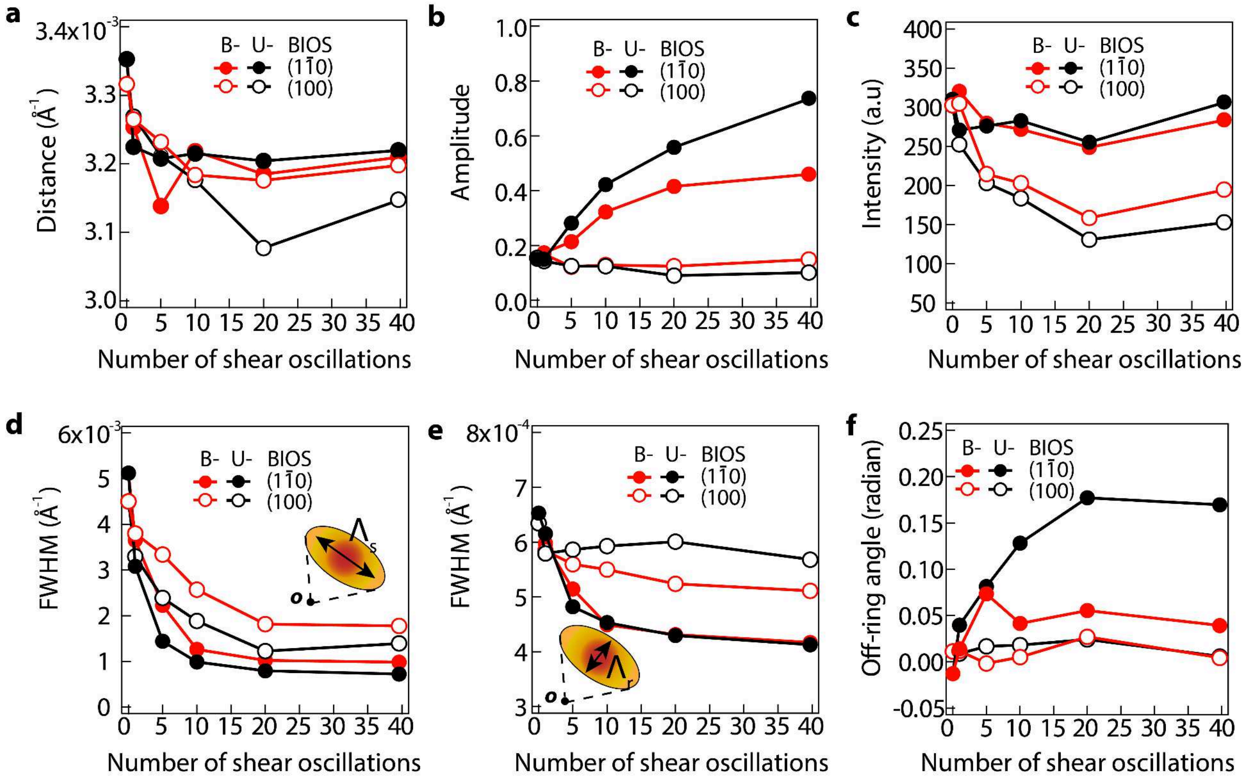

Supplementary Figure 25. Analysis of spots (100) and (1$\bar{1}$0) in SAXS results at normal incidence for samples with increasing numbers of shear oscillations. **a**, Distance of spot centres to origin in reciprocal space, **b**, amplitude of spots, **c**, and integrated intensity. Angular width of spots measured in **d**, azimuthal Λ_s and **e**, radial Λ_r directions. **f**, Angular twist of the elongated spots from the radial direction.

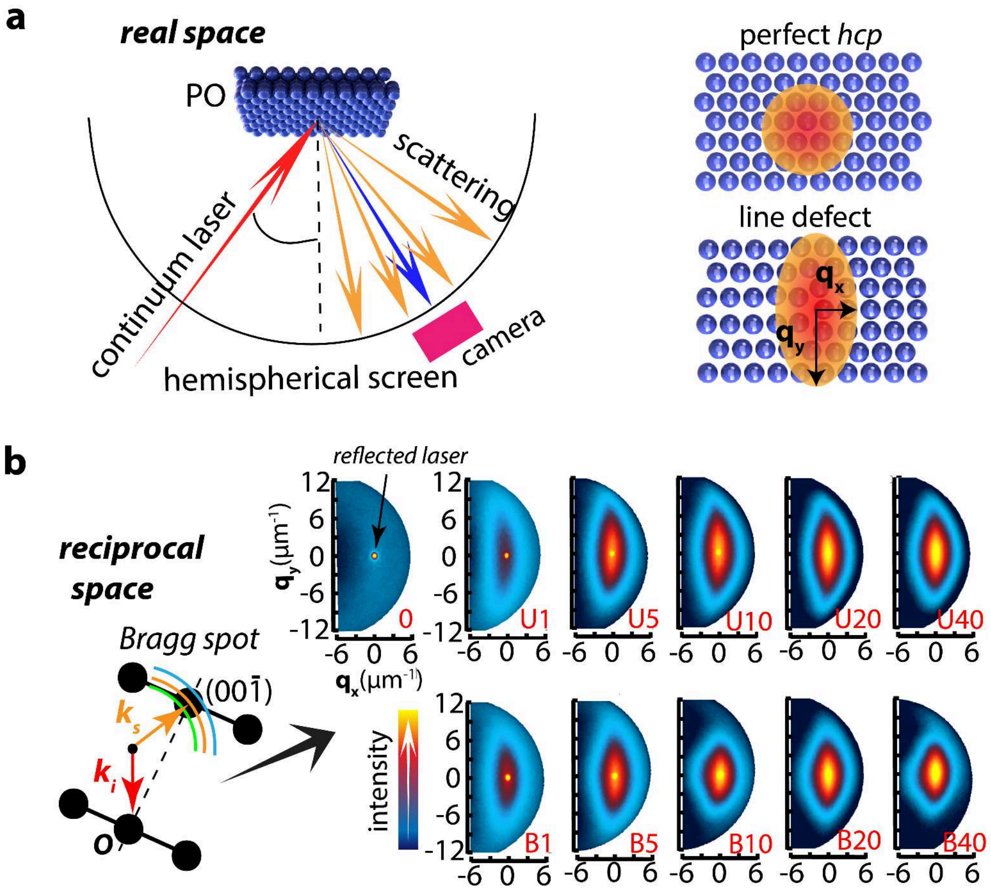

Supplementary Figure 26. Characterisation of 3D reciprocal lattice of PO samples by hyperspectral scattering at all angles. **a**, Left: orientation of hyperspectral scattering measurement, filtered continuum laser incident at 30°, scattered light on hemispherical screen imaged by camera. Right, schematic of how shape of scattering cone (orange) is affected by in-plane defects, $\boldsymbol{q}_{x,y}$ are reciprocal space vectors. **b**, Mechanism and results of measurement: results are slices cut through the centre of the Bragg spot domain. The bright spot in the middle of each image is the specularly reflected laser beam, which is removed in further analysis.

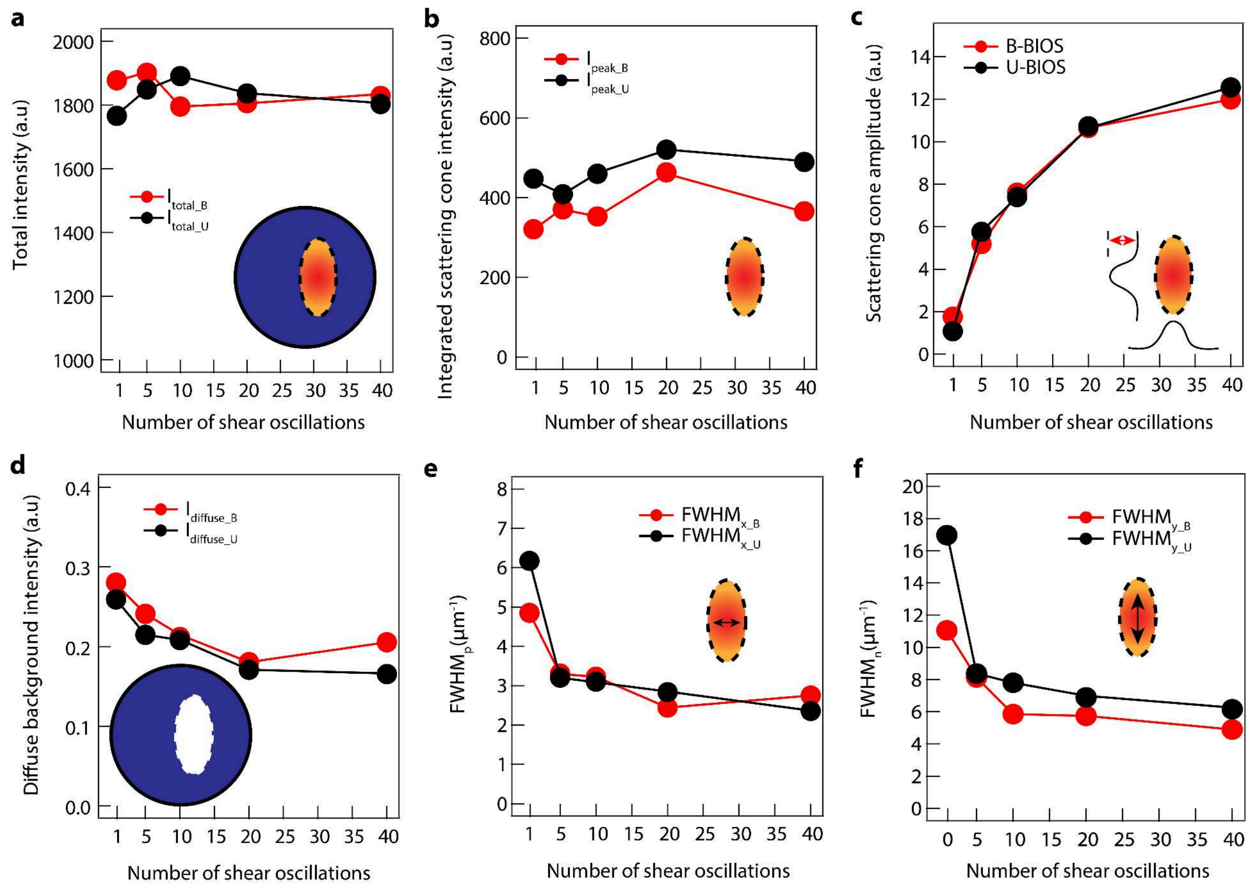

Supplementary Figure 27. Analysis of the optical scattering cones of different PO samples with increasing amount of U-BIOS and B-BIOS. **a**, Integrated intensity of entire scattering. **b**, Integrated intensity of 2D Gaussian-shaped scattering cone. **c**, Amplitude of 2D Gaussian-shaped scattering cone. **d**, Diffuse scattering background outside scattering cone. **e**, FWHM of scattering cone in shearing direction (q_x). **f**, FWHM of scattering cone in q_y-direction in reciprocal space.

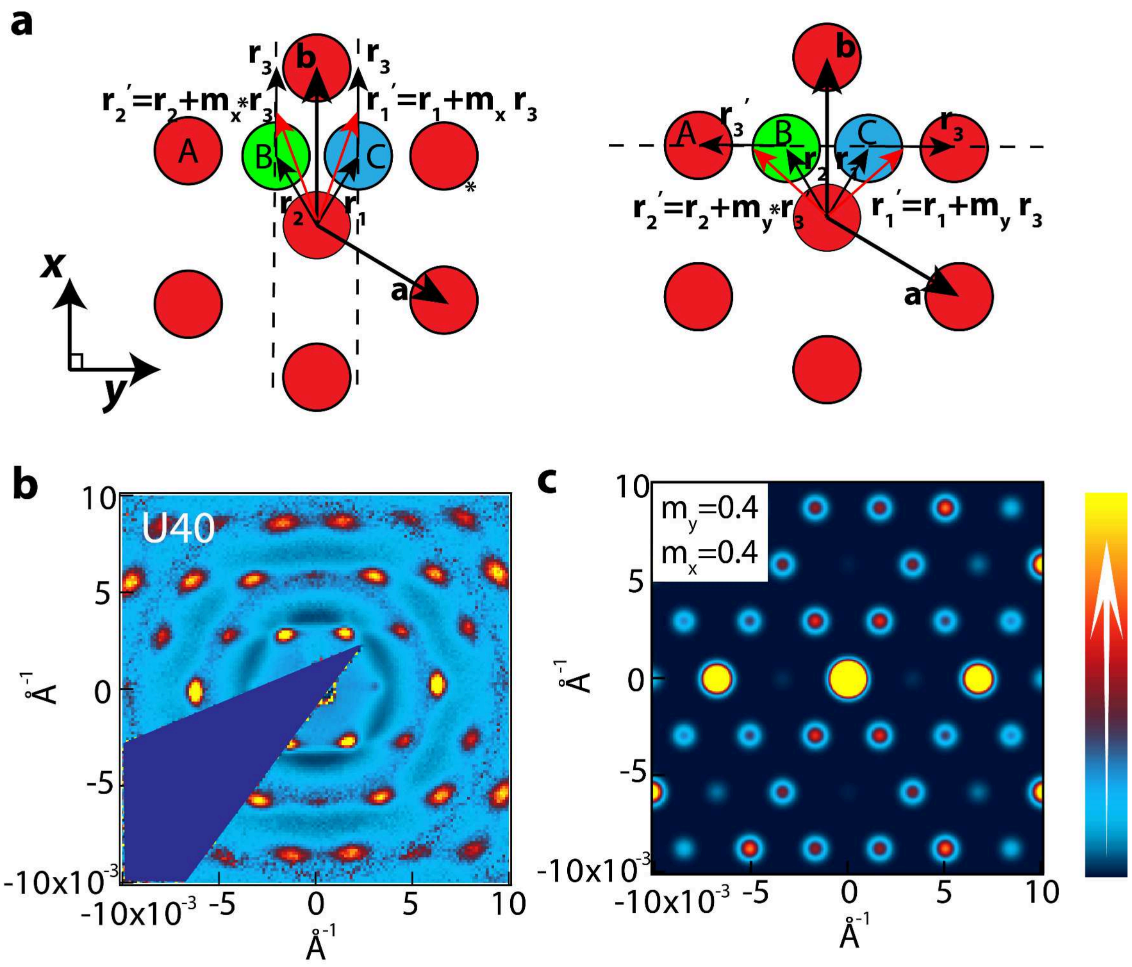

Supplementary Figure 28. Simulation results from sliding layer model compared with experimental results. **a**, Sliding layer model, positions of layers shifted off original registries along *x*- (left) and *y*-directions (right) in real space. A, B and C represent three layers with different translation vectors. **b**, SAXS pattern of sample after 40 passes of U-BIOS at normal incidence after being normalized with diffraction pattern of sample before applying shearing. **c**, Simulated pattern with $m_y = 0.4$ and $m_x = 0.4$ at $q_z = 0$.

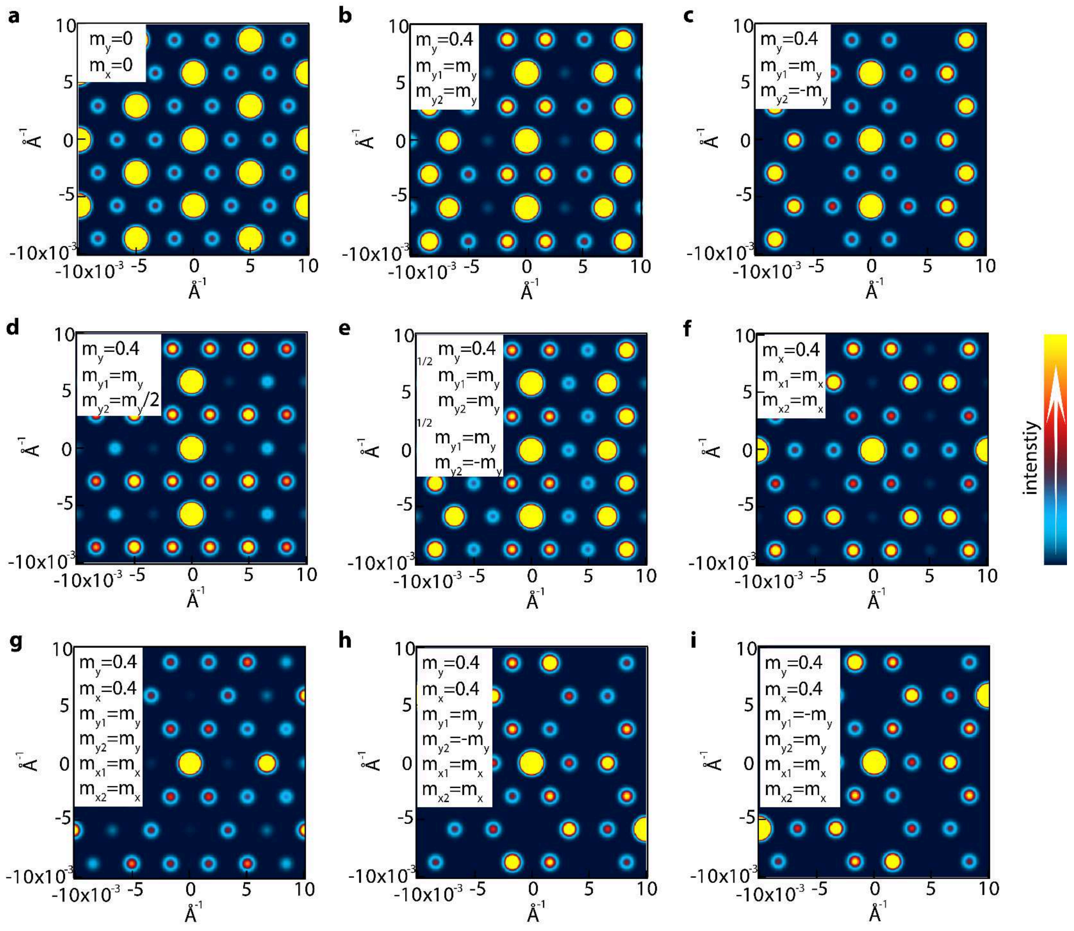

Supplementary Figure 29. Simulation results of SAXS patterns in reciprocal space at $q_z = 0$ with normal incidence and different relative shifts between the *hcp* layers, *x*-direction is along the vertical direction in the real space model. **a**, Ideal *r-hcp* stacking. **b**, Layers shift in opposite y with same $m_y = 0.4$. **c**, Layers shift to $+y$ with same $m_y = 0.4$. **d**, Layers shift in opposite y with different magnitudes. **e**, Superimposed patterns obtained from (b,c). **f**, Layers shift to $+x$ with same $m_x = 0.4$. **g**, Layers shift to $+x$ with same $m_x = 0.4$ and simultaneously to opposite y with same $m_y = 0.4$. **h**, Layers shift to $+x$ with $m_x = 0.4$ and to $+y$ with same $m_y = 0.4$. **i**, Layers shift to $+x$ with $m_x = 0.4$ and to $-y$ with $m_y = 0.4$.

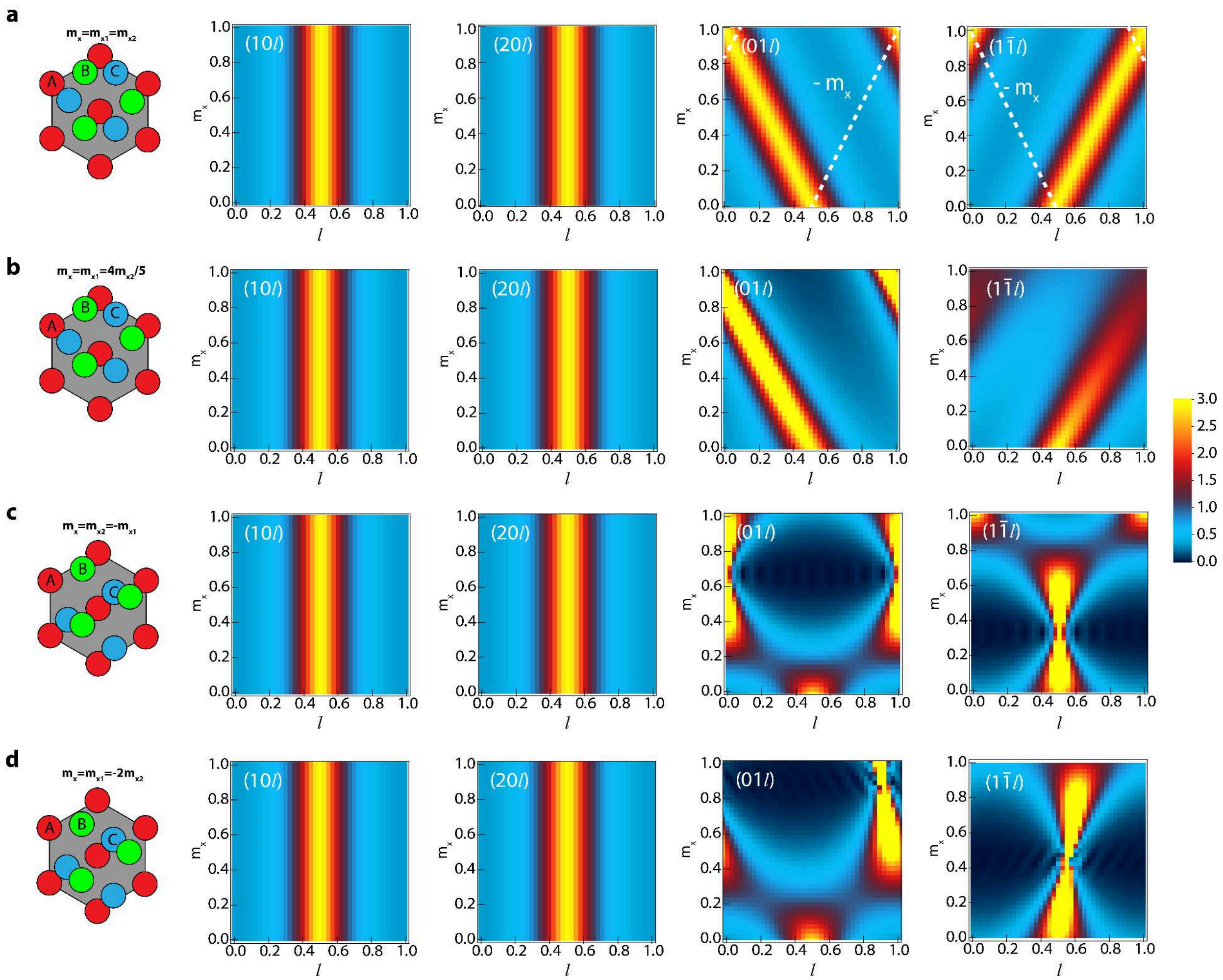

Supplementary Figure 30. Simulation results of X-ray scattering intensity along different rods within one period (horiz-axis) from different configurations of *hcp* layers shifting along x direction only, m_{x1} is the shift of layer C, m_{x2} is the shift of layer B. Scattering intensities along the rods $(10l)$, $(20l)$, $(01l)$ & $(1\bar{1}l)$ when **a**, both B and C layers shift in $+x$ with same magnitude, **b**, both layers shift in $+x$ but one shifts more than the other, **c**, the two layers shift in opposite *x*-directions with same magnitude, **d**, layers shift in opposite *x*-directions with different magnitudes. The scattering distributions of rods ($\bar{1}$0l) and ($\bar{2}$0l) are the mirror of (01l) and (02l), and match the results of (01l) and (02l) when the magnitude m_x is flipped.

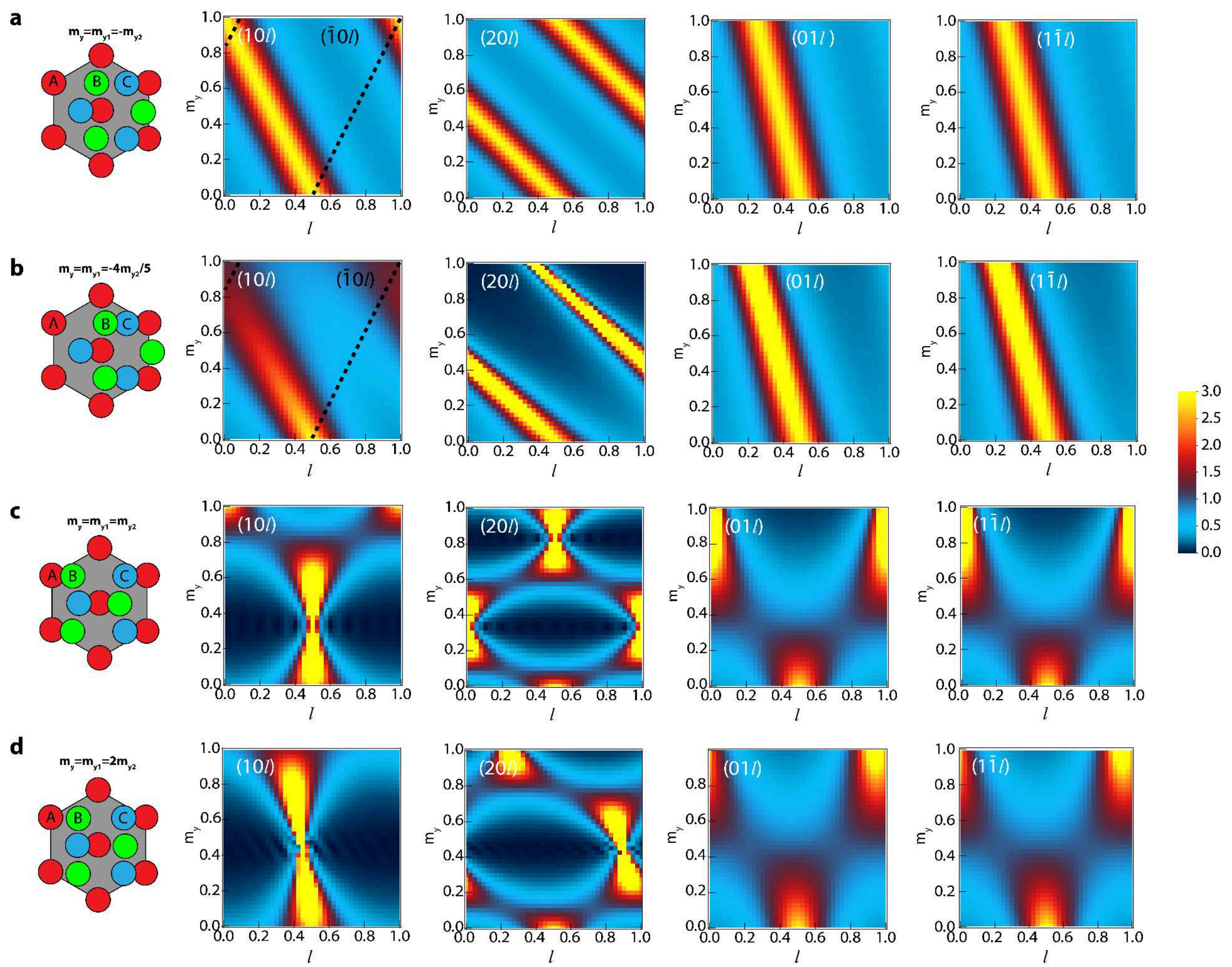

Supplementary Figure 31. Simulation results of X-ray scattering intensity along different rods within one period (horiz-axis) when the *hcp* layers shift in the *y*-direction only, m_{y1} is the shift of layer C, m_{y2} is the shift of layer B. Scattering intensity along the rods $(10l)$, $(20l)$, $(01l)$ & $(1\bar{1}l)$ when **a**, both B and C layers shift in $+y$ with same magnitude, **b**, both layers shift in $+y$ but one shifts more than the other, **c**, the two layers shift in opposite *y*-directions with same magnitude, **d**, Layers shift in opposite *y*-directions with different magnitudes. The scattering distributions of rods $(\bar{1}0l)$, $(\bar{2}0l)$ are the mirror of $(01l)$, $(02l)$, and match the results of $(01l)$, $(02l)$ when the magnitude m_y is flipped.

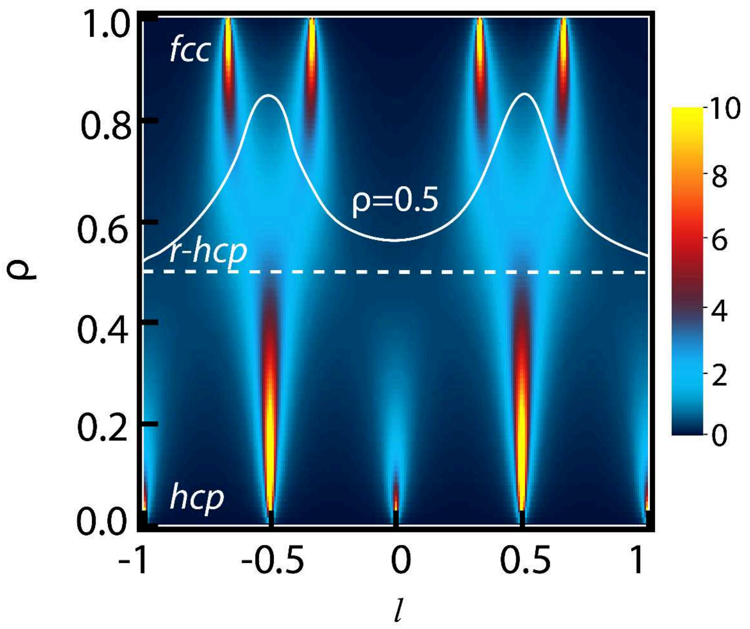

Supplementary Figure 32. Scattering along the rods within two periods in reciprocal space ($l = -1$ to $l = 1$) with different fractions of stacking faults in the stacking sequence of the *hcp* layers. Here ρ is the probability of a layer to have a stacking fault, $\rho = 1$ indicates the stacking is pure *fcc*, $\rho = 0$ indicates the stacking is pure *hcp*, and $\rho = 0.5$ represents completely randomly stacked *hcp* layers (*r-hcp*). The white spectrum is the shape of the scattering intensity distribution along the dashed line.

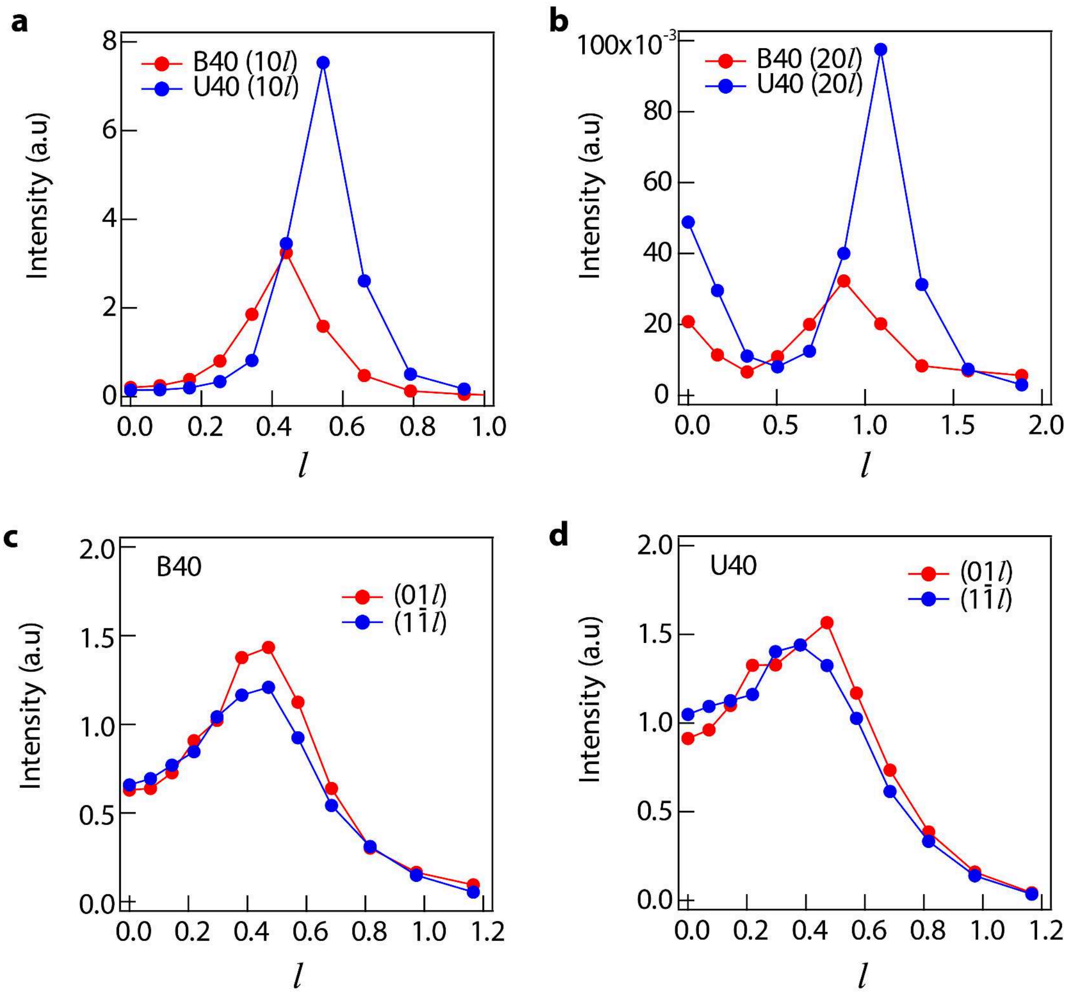

Supplementary Figure 33. X-ray scattering intensity distributions compared along rods of PO samples sheared with 40 passes of B-BIOS and U-BIOS. **a**, Rod $(10l)$ of both samples. **b**, Rod $(20l)$ of both samples. **c**, Rods $(01l)$ & $(1\bar{1}l)$ of sample sheared with 40 passes of B-BIOS. **d**, Rods $(01l)$ & $(1\bar{1}l)$ of sample sheared with 40 passes of U-BIOS.

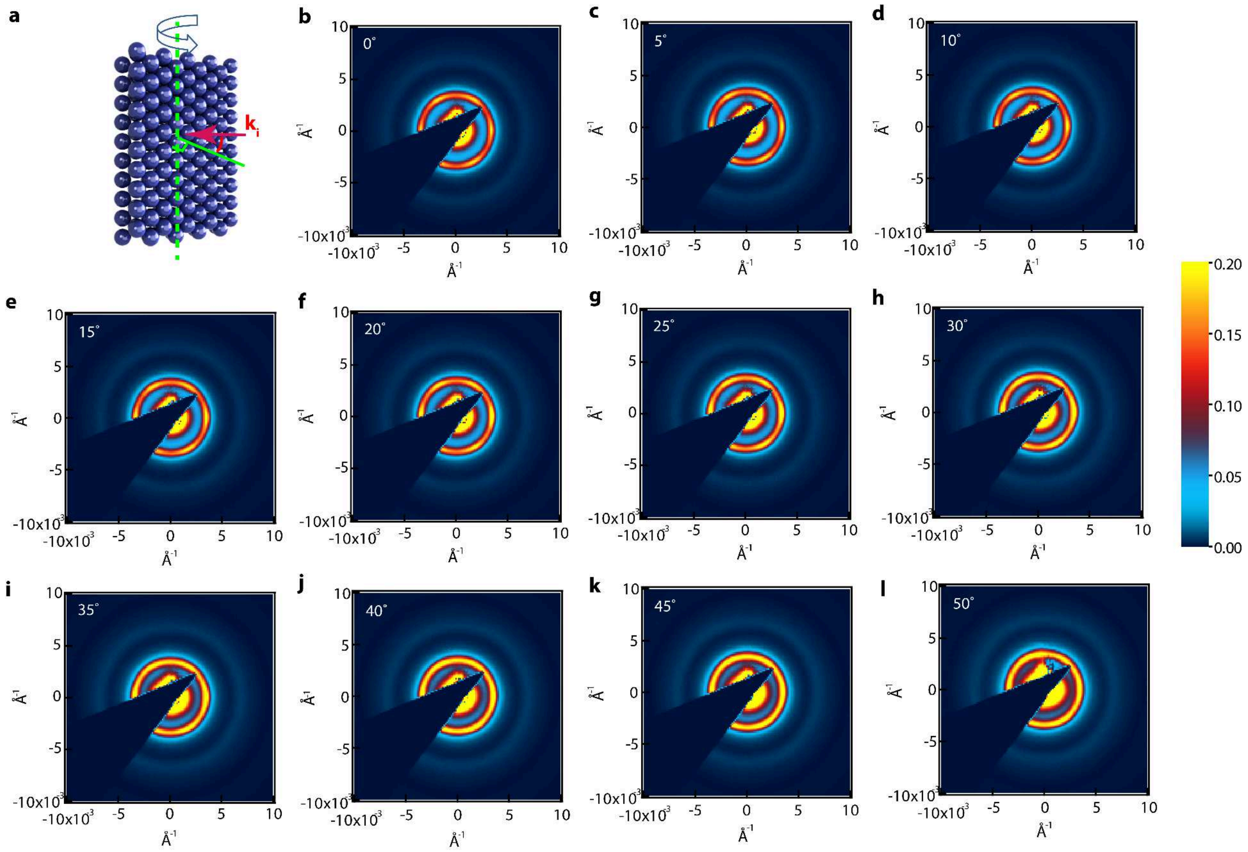

Supplementary Figure 34. SAXS patterns at different incident angles, of sample before applying BIOS. **a**, Experiment geometry, the *x*-direction of the sample is aligned along the vertical direction, the sample is rotated around the vertical axis. **b-l**, SAXS patterns measured at different incident angles varying from 0° to 50° with 5° steps.

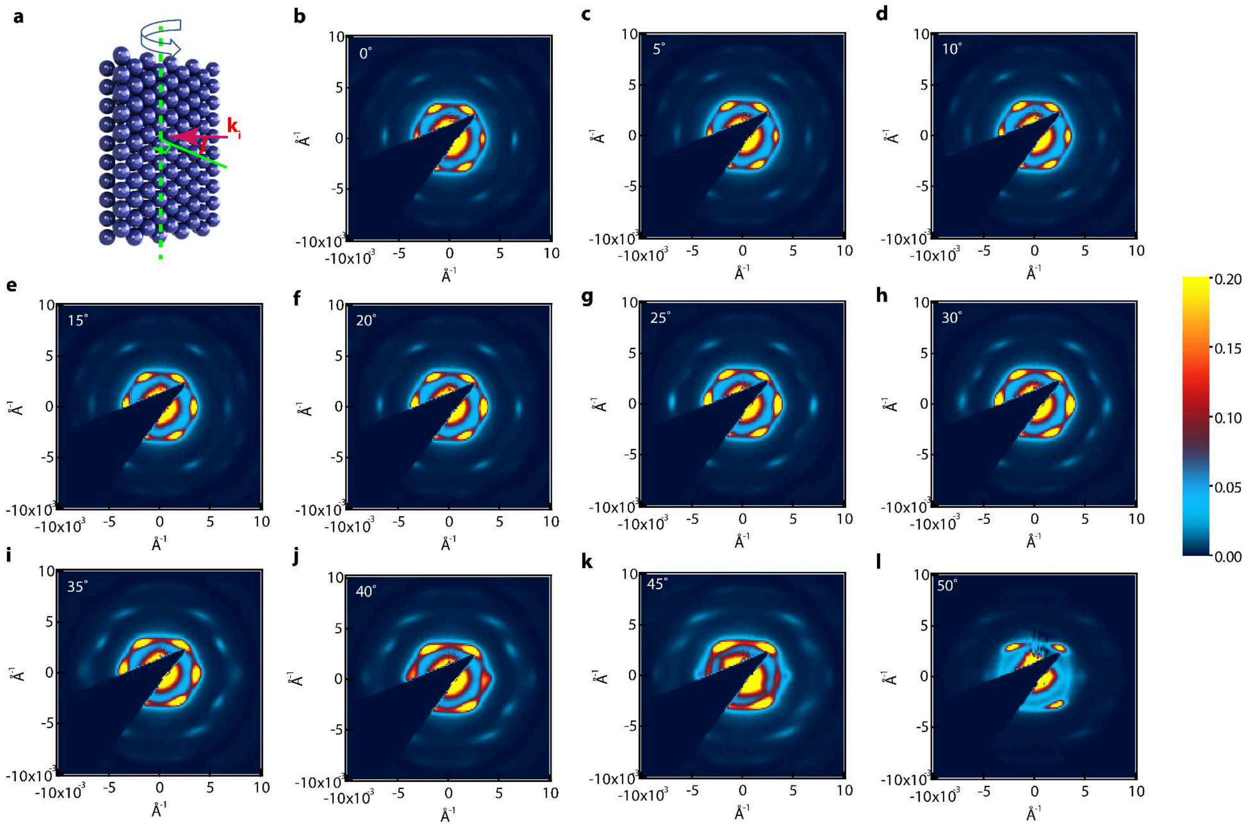

Supplementary Figure 35. SAXS patterns at different incident angles, of sample sheared with 40 passes of B-BIOS. **a**, Experiment geometry, the *x*-direction of the *hcp* planes is aligned along the vertical direction, the sample is rotated around the vertical axis. **b-l**, SAXS patterns measured at different incident angles varying from $0°$ to $50°$ with $5°$ steps.

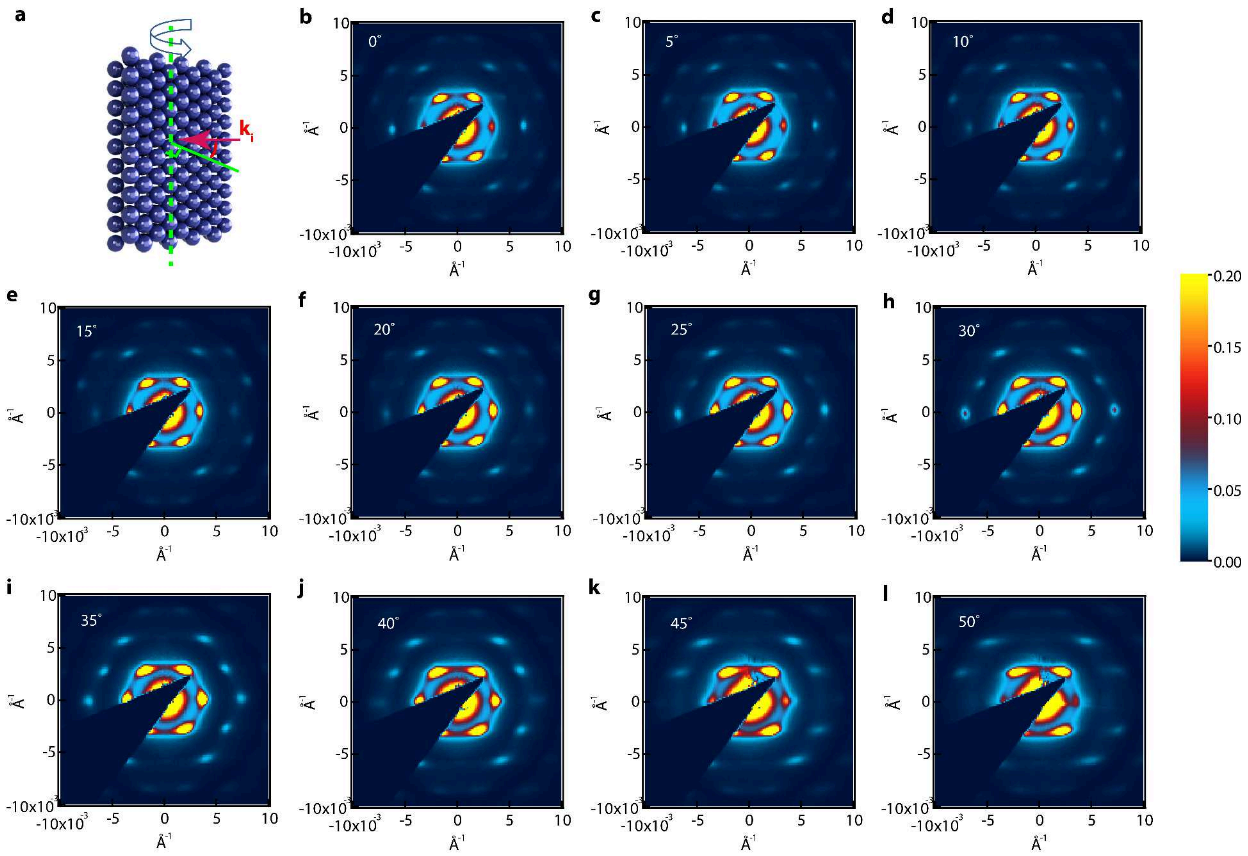

Supplementary Figure 36. SAXS patterns of sample sheared with 40 passes of U-BIOS at different X-ray incident angles. **a**, Experiment geometry, the *x*-direction of the *hcp* planes is aligned along the vertical direction, the sample is rotated around the vertical axis. **b-l**, SAXS patterns measured at different incident angles varying from 0° to 50° with 5° steps.

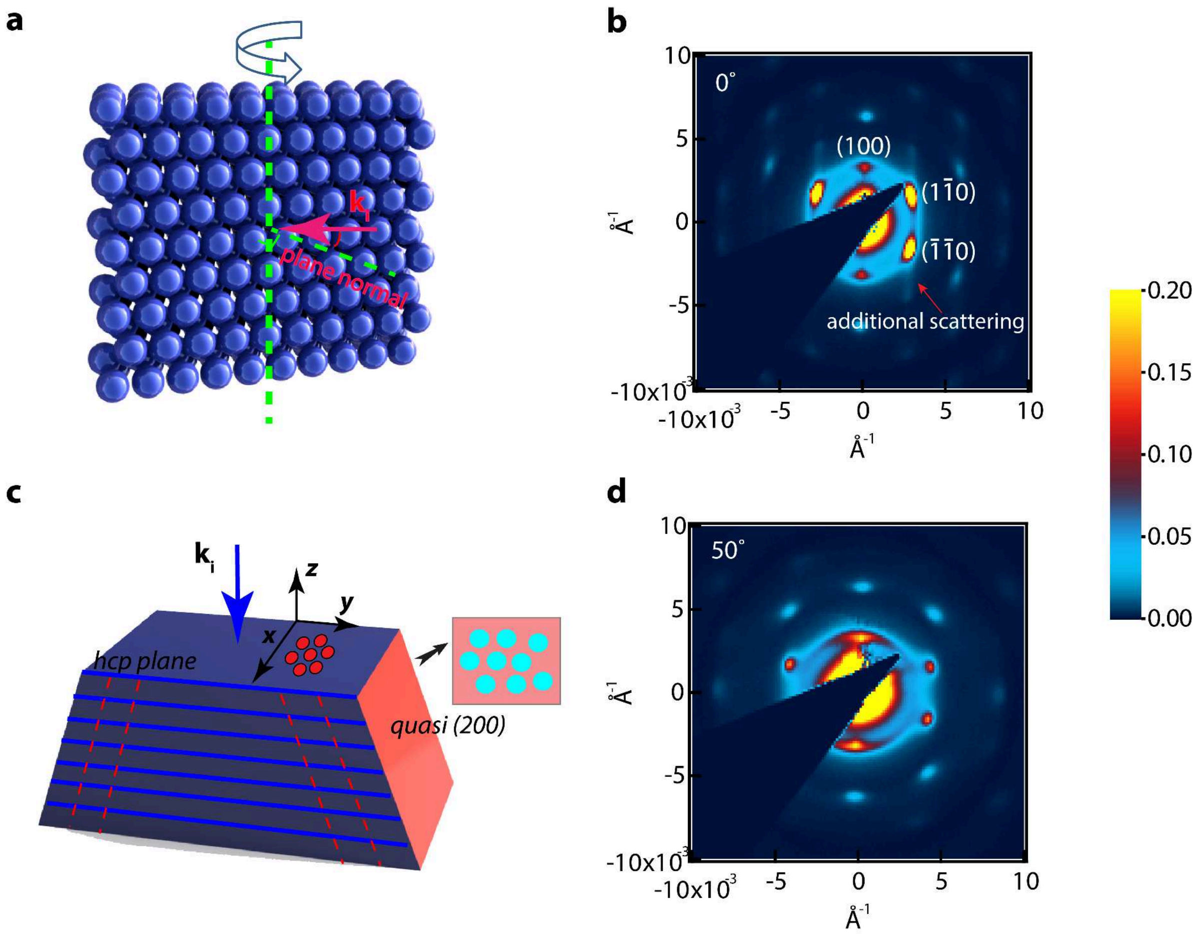

Supplementary Figure 37. SAXS patterns of PO sample with 40 passes of U-BIOS, at different X-ray incident angles with *y*-direction as the vertical axis rotating axis. **a**, Measurement geometry, sample is rotated around *y*-direction. **b**, SAXS pattern of sample at normal incidence, red arrow indicates the possible additional scattering from the quasi-(200) planes. **c**, Illustration of relative positions of different planes in the measurement, blue represents *hcp* planes parallel to the surface, red represents quasi-(200) planes which are at $\sim 35°$ with respect to the normal direction of the *hcp* planes. **d**, SAXS pattern of the sample tilted by $50°$.

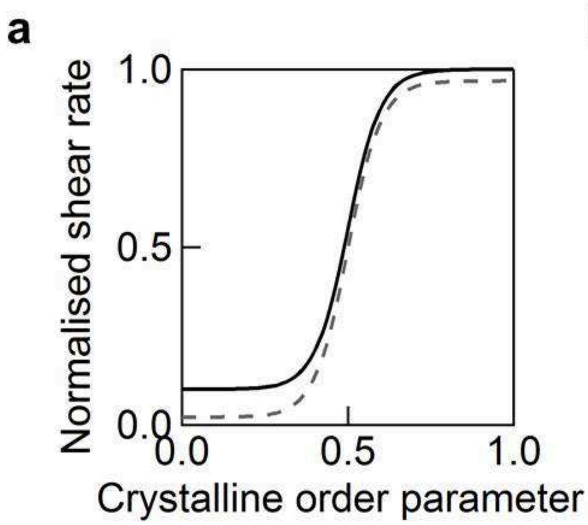

Supplementary Figure 38. **a**, Transfer function from two phase model, with amorphous to crystalline transition at specific depth H. **b**, Resulting nonlinear diffusion of crystal order into sample, showing depth transition which exactly matches Eqn (10).

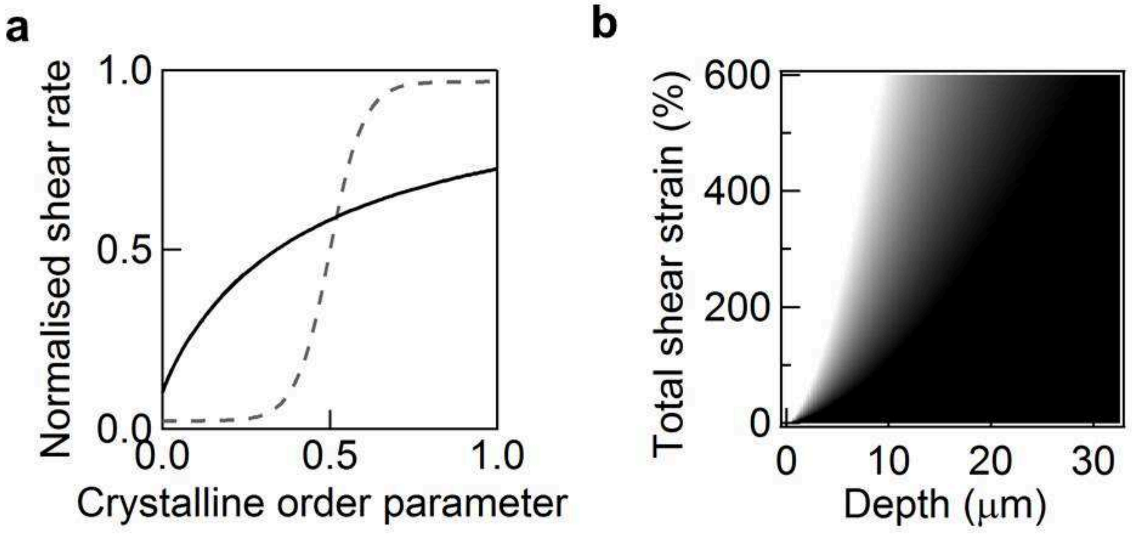

Supplementary Figure 39. **a**, Transfer function from full model (solid). **b**, Resulting nonlinear diffusion of crystal order into sample, showing gradual depth transition which matches experiment.

Supplementary discussions

Discussions on Abaqus simulations Abaqus simulations are performed with a 2D model (Supplementary Figure 4, Supplementary Movies 1-2), and results with different R_E are shown in order to evaluate the effects of the relative rigidity between PET and PO, where $R_E = E_{\text{PET}}/E_{\text{PO}}$, and E_{PET} and E_{PO} are the Young's moduli of PET and PO respectively. The distinct mechanical properties of PET and PO layers give significant discontinuity in the axial normal strain distribution across the thickness of the beam (Supplementary Figure 7). Our results confirm our expectation that shear strain reduces significantly when R_E gets smaller, but when $R_E = 240$, there is only pure stretching or pure compressing in the PET layers (Supplementary Figure 4c). The result with $R_E = 1000$ agrees well with our calculation and experimental strain measurement (Supplementary Figure 5). The simulation result only shows data up to a $\phi = \pm\frac{\pi}{3}$ due to the limitations of the model. A rather long beam needs to be used in the simulation in order to avoid excessive distortion of the elements at the free ends during the bending process. Thus even if the contact angle has reached π, the rest of the film deflects in the opposite direction of bending so as to release the strain in the ends. As a result the shear strain starts to decrease even within the contacted area when $\phi \approx \frac{\pi}{4}$ (depending on R_E) before it reaches the expected maximum amplitude. This accounts for why the simulated ε_{xz} curves drop before $\phi = \frac{\pi}{2}$.

In addition, the PO layer is significantly compressed in the thickness direction right at the top of the cylinder ($\phi = 0$, where ϕ is the position angle), which gives a ε_{zz} (also shown in the width of the discontinuities in Supplementary Figure 4c). This is due to the use of an isotropic hyperelastic model for PO in the simulation, whilst in reality the material is strongly anisotropic. The current model is adopted simply because of the stability of the simulation, and also due to the extreme difficulty in characterising the mechanical properties in the thickness direction of the film. After correcting ε_{xz} for the true distance, it turns out that this does not affect the accuracy of the simulation. To avoid misleading readers that the shearing is not purely parallel to the surface of the layers and that it should contain a compression induced flow, we show optically that the colour of the PO does not change in bending (see Figure 4), which indicates that there is no obvious strain

in the thickness direction.

Discussions on the effects of the exponential variations in layer spacing with depth Supplementary Figure 21a shows the experimental result of the spatial frequency of the layered structure in the depth direction of PO samples with 40 passes of B-BIOS. The slope of the red section of the spatial frequency of the sample (from 0 μm to 10 μm) is relatively steep, but then it reaches a plateau moving into the green section (> 10 μm). The deepest-z data point shows much increased noise which comes from the blurring of the images by poor staining as well as the increasing disorder.

Supplementary Figure 21c shows the reflectance spectra of two samples fabricated both with 20 passes of U-BIOS at room temperature. Double-peak features exist in both samples, with a green peak at short wavelengths ($F \approx 5.52$) and a red peak at longer wavelengths ($F \approx 5.22$). The two spectra are typical for samples sheared at room temperature. Positions of the two peaks may vary between the two positions ($5.22 < F < 5.52$), with relative intensity of the peaks varying randomly between samples. Using FFT analysis of a spatially modulated model built according to the fitted exponential spatial distribution function from experimental data shows clearly that there are two main frequencies in the spatial period of the layered structure (Supplementary Figure 21d), which matches the double-peak positions in Supplementary Figure 21c very well. By mapping the frequencies from the FFT analysis to the experiment results, we show that the green peak in the double peak feature in Supplementary Figure 21c corresponds to layers whose spacings are within the plateau section of the exponential curve (in green background colour) of the sample (Supplementary Figure 21a), while the red peak corresponds to layers within the depth section in the red background colour. We compare the normalized reflectance of the sample with 40 passes of B-BIOS from measurement with simulated results using the transfer matrix method (TMM). The model used in TMM simulations exactly uses the exponential fit to the structure periodicity in Supplementary Figure 21a and the changes in $\Delta n_{eff}(z)$ in Supplementary Figure 21b. A sharp and intense peak at shorter wavelengths (555 nm) and a low intensity peak at longer wavelengths (605 nm) are observed in the experimental results, which correspond to the FFT central frequencies of

the red and green parts of the sample in Supplementary Figure 21a. Slight differences in shape and peak position between the simulation and experimental results are observed (Supplementary Figure 21e). However any differences can be accounted for by using a slightly modified model, shown in Supplementary Figure 21f, so that the peak position matches well with the experimental data, and the double-peak feature has disappeared. In this slightly modified model the frequency of the green part of the sample is reduced by $\Delta F = 0.08$, which is well within the error of our structural data. The above results show that the number and positions of the optical reflection peaks depend on the number of strong frequencies of spatial variation within the layers. The shape of the spectra thus depend on the interplay between the frequency distributions in the red and green parts of the sample in Supplementary Figure 21a. The intensity of the calculated peak is higher than in experiment most likely due to the various disorders between the layers which are suppressed in our FFT process, and the in-plane disorder of the layers, which is not included in the simulations. TMM simulations show that for a fixed number of layers, for example 80 layers, the amplitude of the reflection is almost linear with Δn_{eff} (Supplementary Figure 22).

Discussions on the anomalous X-ray scattering along the Bragg rods in POs with the sliding layer model simulations Supplementary Figure 33 shows the scattering intensity along the rods in reciprocal space, which is obtained by extracting the center intensity of the spots from goniometer SAXS measurements with incident angle varying from $0° - 50°$ in $5°$ steps (Supplementary Figure 35,36). Although integrated intensities over the cross-section of the rods would best be used, this is impossible in such gonio-measurements since the imaging plane cuts the rods at large tilt angles. We use here the amplitude of the spots, which is the square of the integrated intensity. We also account for errors due to the limited pixel size of the detector.

Peaks in the measured scattering distributions along rod (10) & rod (20) of PO samples with 40 passes of U-BIOS are much sharper and stronger than those in the sample with 40 passes of B-BIOS (Supplementary Figure 33a & b). The positions of the peaks shift from the ideal positions of $l = 0.5$ either slightly (Supplementary Figure 33a) or enormously (Supplementary Figure 33b), which agrees well with our experimental results measured with incident beam parallel

to the surface of the samples. According to the sliding layer model (Supplementary Figure 28), we propose that the shifts are possibly due to a relative horizontal movement between the *hcp* layers inside the sample. Since the shifts of the layers in the *x*-direction alone do not affect the intensity distributions along rods (10) and (20) according to simulation results (Supplementary Figure 30), the measured scattering along the two rods must be modified by shift components of the layers in the *y*-direction (Supplementary Figure 34). There are two modes of shifting in the lateral (y) direction: shifting of both layers B and C in the same direction, and shifting of layers B and C in opposite directions (possibly both exist in the samples). The simulation results (Supplementary Figure 31) show that when layer B and layer C shift in opposite directions sideways ($m_y = m_{y1} = m_{y2}$), scattering is mainly confined to the middle position of rod (10) and the nodes of rod (20) as long as the shift is not too large ($m_y < 0.6$). This matches the shapes of the intensity distributions of rods (10) and (20) of both samples. The peaks of the distribution get sharper and stronger when the magnitude of the relative shift increases within a moderate range ($m_y \leq 0.4$), which explains why the peaks along rods (10) (20) of U-BIOS sample are much more intense and sharper than those of the B-BIOS sample, assuming that relative shifts between layers are in general larger in U-BIOS samples than in B-BIOS samples ($m_{y-B} < m_{y-U}$). However, it does not explain the shift of the peak positions off the theoretical positions ($l = 0.5$). In order to explain this phenomenon, asymmetric movements in the lateral direction of the two layers are necessary to introduce. For example both layers B and C shift collectively to one direction ($m_y = m_{y1} = -m_{y2}$), or they shift in opposite directions but with different magnitudes of shift ($m_y = m_{y1} = 2m_{y2}$). Scattering along rods (01) and ($1\bar{1}$) shows similar shapes for both samples (Supplementary Figure 33c & d): the maximum is half way between nodes along the rod with a slight shift to low m, with scattering near the origin of the rod much higher than the other end. Intensities of the scattering along rods (01) & ($1\bar{1}$) are lower than those of rods (10) & (20). We note that the difference between scattering intensities of rods (10) & ($1\bar{1}$) from samples with U-BIOS and those from sample with B-BIOS near the base of each rod. The scattering intensity from the U-BIOS samples here is twice that of the B-BIOS samples, however the position and intensity of the peaks n the scattering curve remain almost the same. There might be two possible explanations for this: the first is

the difference between the magnitudes of sliding layer shifts in the *x*-direction, which strongly changes scattering along rods (01) and $(1\bar{1})$ (Supplementary Figure 30); the other reason might be the existence of quasi-(200) planes in the U-BIOS samples. As shown in Supplementary Figure 37, there is extra scattering between the spots along the direction parallel to $\boldsymbol{a}^*$ in the normal incidence SAXS pattern of samples with U-BIOS, which probably comes from scattering off quasi-(200) planes. The orientation of the quasi-(200) planes is close to the normal direction of the surface *hcp* planes of the sample, therefore when the sample is illuminated from a normal direction, extra scattering generated by the stacking sequence order of these planes could add to the SAXS pattern. Since quasi-(200) planes are only found in U-BIOS samples with a preferred orientation, no extra scattering is observed in the diffraction patterns of B-BIOS samples. It is assumed that by rotating the U-BIOS samples around the *y*-direction axis normal incidence diffraction pattern of the quasi-(200) planes can be observed. However, in experiment it is extremely difficult to distinguish the scattering pattern from the quasi-(200) planes from the image when the sample is rotated to high angles ($\sim 55°$) because, firstly, signals from the quasi-(200) planes are weak due to their poor structural order compared to the surface *hcp* planes, and secondly, scattering patterns from the surface *hcp* planes get stretched and their intensity grows stronger at high angles due to the increase of the amount of crystal illuminated (Supplementary Figure 37d). Therefore signals of the quasi-(200) planes overlap with those from tilted *hcp* planes, and it is hard to extract useful signals buried underneath.

Discussions on the model for shear ordering of nanoparticles We model the composite as layers which when sheared, locally apply forces that lead to enhanced crystal order (as in Figure 8a). The different types of order, which range from the stacking of planes to the nanoparticle ordering within each plane, are not distinguished in this model, and all lateral inhomogeneity is ignored. The viscosity of each layer is dependent on the local crystal order, $c_i(z_i, t)$, at time t and depth z_i within the composite, and decreases as the order increases. Since the shear rate within each layer $\dot{\gamma}_i$ depends inversely on the viscosity we parametrise it as

$$\dot{\gamma}_i = a \ f(c_i) \tag{1}$$

where $f(c)$ is a monotonically increasing transfer function dependent on the local crystal order, and $a(t)$ is a global normalisation constant to be found below.

When additional shear strain $d\gamma_{\text{tot}}$ is applied in time dt to the entire composite of total thickness h, it is distributed among the stack of layers according to the local viscosities and hence shear rates. Hence $h\, d\gamma_{\text{tot}} = \sum_i \Delta\, d\gamma_i$ summed over the layers of individual thickness Δ or equivalently

$$h\, d\gamma_{\text{tot}} = \int_0^h dz\ (\dot{\gamma}_i dt) \tag{2}$$

This defines the normalisation condition a, so that

$$a(t) \int_0^h dz\ f[c(z,t)] = h\, \frac{d\gamma_{\text{tot}}}{dt} \tag{3}$$

is then constant at each time step.

The forces between two neighbouring layers leading to improved order are (in each time step) set by the difference of their local velocities producing an impulse, $F_i\, dt \propto (v_i - v_{i-1})$. Since $v_i \propto \dot{\gamma}_i$ we can parameterise the improvement in crystalline order in each time step,

$$dc_i = u\, dt(\dot{\gamma}_i - \dot{\gamma}_{i-1}) \tag{4}$$

or generalising to continuous coordinates,

$$\frac{\partial c}{\partial t} = u\frac{\partial \dot{\gamma}}{\partial z} = u\, a(t)\, \frac{\partial f}{\partial z} \tag{5}$$

This set of equations (1),(3), and (5) form a complete description of the dynamics under shear, and is easily simulated for different dependences of the viscosity on the crystal order.

For the case where only two phases can exist, amorphous (a) or ordered (x), the transfer function $f(c)$ becomes a simple step function (Figure 8b). In that case the model can be compared to a simple analytic solution introduced in Sherada et al., (Phys. Rev. Lett. vol. 105, p.228302 (2010)), with a sharp interface between the two phases at depth H and a ratio between the viscosities of these phases of $M' = M + 1$. In this previous situation, the movement of the interface H

was assumed to be dependent on the shear rate in just one of the phases, but this leads to essentially the same result, as we compare below. The equations under these assumptions simplify to

$$\begin{aligned} dH &= u\dot{\gamma}_a & (6) \\ hd\gamma_{\rm tot} &= H\dot{\gamma}_x + (H-h)\dot{\gamma}_a & (7) \\ \dot{\gamma}_x &= M'\dot{\gamma}_a & (8) \end{aligned}$$

which give

$$\int dH \left[1 + M\frac{H}{h}\right] = u \int d\gamma_{\rm tot} \quad (9)$$

The resulting quadratic can be solved to yield the dependence of the boundary on applied shear,

$$H(\gamma_{\rm tot}) = \frac{h}{M}\left\{\sqrt{1 + 4uh\gamma_{\rm tot}/M} - 1\right\} \quad (10)$$

This is plotted in Supplementary Figure 38 together with the full solution from the continuum model using the step transfer function $f(c)$, showing their full agreement.

When the transfer function is not as steeply dependent on a critical crystal order, a different behaviour is seen (Supplementary Figure 39).
